# Cameroonian blackflies (Diptera: Simuliidae) harbour a plethora of RNA viruses

**DOI:** 10.1093/ve/veaf024

**Published:** 2025-04-05

**Authors:** Lander De Coninck, Amber Hadermann, Ludovica Ingletto, Robert Colebunders, Kongnyu Gamnsi Njamnshi, Alfred Kongnyu Njamnshi, John L Mokili, Joseph Nelson Siewe Fodjo, Jelle Matthijnssens

**Affiliations:** Department of Microbiology, Immunology and Transplantation, Rega Institute, Laboratory of Viral Metagenomics, KU Leuven, Herestraat 49 box 1040, Leuven 3000, Belgium; Global Health Institute, University of Antwerp, Campus Drie Eiken, Gouverneur Kinsbergencentrum, Doornstraat 331, Antwerp 2610, Belgium; Department of Microbiology, Immunology and Transplantation, Rega Institute, Laboratory of Viral Metagenomics, KU Leuven, Herestraat 49 box 1040, Leuven 3000, Belgium; Department of Medical and Surgical Sciences (DIMEC), University of Bologna Via Giuseppe Massarenti 9, Bologna 40138, Italy; Global Health Institute, University of Antwerp, Campus Drie Eiken, Gouverneur Kinsbergencentrum, Doornstraat 331, Antwerp 2610, Belgium; Department of Tropical Disease Biology, Liverpool School of Tropical Medicine, Pembroke Place Liverpool, Liverpool L3 5QA, United Kingdom; Brain Research Africa Initiative (BRAIN), Nsimeyong II P.O. Box 25625, Yaoundé, Cameroon; Brain Research Africa Initiative (BRAIN), Nsimeyong II P.O. Box 25625, Yaoundé, Cameroon; Department of Biology, Viral Information Institute, San Diego State University, 5500 Campanile Drive, San Diego, CA 92182, United States; Global Health Institute, University of Antwerp, Campus Drie Eiken, Gouverneur Kinsbergencentrum, Doornstraat 331, Antwerp 2610, Belgium; Department of Microbiology, Immunology and Transplantation, Rega Institute, Laboratory of Viral Metagenomics, KU Leuven, Herestraat 49 box 1040, Leuven 3000, Belgium

**Keywords:** blackflies, Simuliidae, virome, RNA viruses, onchocerciasis

## Abstract

Strong epidemiological evidence suggests that onchocerciasis may be associated with epilepsy—hence the name onchocerciasis-associated epilepsy (OAE). However, the pathogenesis of OAE still needs to be elucidated, as recent studies have failed to detect *Onchocerca volvulus* in the central nervous system of persons with OAE. Therefore, it was suggested that a potentially neurotropic virus transmitted by blackflies could play a role in triggering OAE. To investigate this hypothesis, adult blackflies were collected in an onchocerciasis-endemic area with a high OAE prevalence in the Ntui Health District, Cameroon. A viral particle-based shotgun sequencing approach was used to detect viral sequences in 55 pools of 10 blackflies. A very high abundance of viral reads was detected across multiple (novel) viral families, including viral families associated with human disease. Although no genomes closely related to known neurotropic viruses were found in the blackfly virome, the plethora of novel viruses representing novel species, genera and even families warrant further exploration for their potential to infect vertebrates. These results could serve as a first step for studying the viruses associated with the haematophagous blackfly, which also could be present in their nematode host *O. volvulus*. Exploring the diversity of viruses in blackflies should be included in the active surveillance of zoonotic diseases.

## Introduction

Human onchocerciasis, commonly known as river blindness, is a parasitic disease caused by the filarial worm *Onchocerca volvulus* (Nematoda: Secernentea; Spirurida; Filariidae; Onchocerca). Transmission of the parasite occurs through repeated bites of female blackflies belonging to the *Simulium* species (Brattig et al. 2021). Onchocerciasis is classified as one of the neglected tropical diseases recognized by the World Health Organization.

Onchocerciasis-endemic regions with high ongoing or past *O. volvulus* transmission are known to have high epilepsy prevalence (Pion and Boussinesq 2012; Levick et al. 2017; Colebunders et al. 2018b; Siewe Fodjo et al. 2018; Boullé et al. 2019; Mukendi et al. 2019; Raimon et al. 2021). While onchocerciasis is commonly thought to affect only the skin and eyes, emerging epidemiological evidence suggests a potential direct or indirect association between *O. volvulus* infection and epilepsy, giving rise to the term ‘onchocerciasis-associated epilepsy’ (OAE), also known as ‘river epilepsy’ (Chesnais et al. 2018, 2020; Colebunders et al. 2019, 2021; Hadermann et al. 2023). OAE presents with a wide spectrum of epileptic seizures, including generalized tonic–clonic seizures, myoclonic seizures, absence seizures, and head nodding seizures (Colebunders et al. 2018a). Nodding and Nakalanga syndromes are considered to be phenotypic presentations of OAE (Colebunders et al. 2018a, Van Cutsem et al. 2023) Nakalanga syndrome is characterized by severe growth retardation, delayed sexual development, mental impairment, facial deformation, kyphoscoliosis, and epileptic seizures (Föger et al. 2017, Siewe Fodjo et al. 2019).

So far, the pathogenesis of OAE is not yet established. Prior to the introduction of ivermectin mass drug distribution, *O. volvulus* microfilariae (mf) had been detected in the cerebrospinal fluid (CSF) of individuals with onchocerciasis (Duke et al. 1976). However, in more recent studies, neither *O. volvulus* mf nor DNA could be detected in CSF (Winkler et al. 2013, Hotterbeekx et al. 2020) nor in postmortem brain tissue of persons with OAE (Hotterbeekx et al. 2019). Therefore, it was suggested that a potentially neurotropic virus transmitted by blackflies, or an endosymbiont of the *O. volvulus* parasite, could play a role in the pathogenesis of OAE (Colebunders et al. 2014, Hadermann et al. 2023). In 2022, Edridge and colleagues discovered a divergent rhabdovirus in the bloodstream of a 15-year-old girl with nodding syndrome from Mundri West Country in South Sudan (Edridge et al. 2022). They named this virus ‘Mundri virus’ (MUNV) and classified it as a novel virus. Subsequent phylogenetic analysis revealed that MUNV belongs to the monophyletic clade of tibroviruses within the family *Rhabdoviridae*. The *Tibrovirus* genus is part of a larger phylogenetic group of arthropod-borne rhabdoviruses. Several tibroviruses have been isolated from biting midges (*Culicoides* spp.) and cattle, and some instances of human detection have been reported (Cybinski et al. 1980, Cybinski and Gard 1986, Gibbs et al. 1989, Grard et al. 2012, Stremlau et al. 2015). In addition, generating more interest for this hypothesis, we recently also showed that viruses of parasitic nematodes can elicit antibody responses in the hosts of the nematodes (Quek et al. 2024).

Very little is known about the virome of blackflies. Kraberger *et al*. described the ssDNA virome of blackflies in New Zealand and reported high numbers of *Genomoviridae*, *Circoviridae*, and *Microviridae* members (Kraberger et al. 2019). Moreover, invertebrate iridoviruses (family *Iridoviridae*) have been identified in blackfly larvae across the globe (Piégu et al. 2013, 2014a, 2014b). In addition, the most studied arbovirus transmitted by blackflies is vesicular stomatitis virus (VSV, family *Rhabdoviridae*; genus *Vesiculovirus*), which typically infects cattle, horses, and swine (Howerth et al. 2002). Nonetheless, there have been no reports of blackflies transmitting arboviruses to humans to date. However, considering the 1700 *Simulium* species described worldwide, it is plausible that blackflies may transmit unidentified viruses from human and zoonotic origins. The aim of this study was to identify potential neurotropic viruses (in this study defined as those related to viruses known to cause infections in the central nervous system, like West Nile virus, Japanese encephalitis virus [*Flaviviridae*], and rabies virus [*Rhabdoviridae*]) transmitted by blackflies, which could play a role in the pathogenesis of OAE.

## Materials and methods

### Sample collection

Adult blackflies investigated were collected in the village of Nachtigal (coordinates of the breeding site: N 4°21.146, E 11°37.953; Garmin GPSMAP78). The gathering took place from 7 a.m. to 6 p.m. over three consecutive days in July 2021. Nachtigal is a rural community of the Ntui Health District, an onchocerciasis-endemic area with a high prevalence of OAE (Siewe Fodjo et al. 2022), in the forest-savannah transition zone in the Centre Region of Cameroon. This community lives close to the Nachtigal rapids on the Sanaga River, which constitutes one of the main sources of blackfly vectors in Cameroon (Siewe Fodjo et al. 2022). The human landing catch method was used to collect the blackflies, and mouth aspirators were used to aspirate blackflies into an empty container every hour (Hendy et al. 2021). At the end of each catching day, blackflies were stored dry at −20°C in the BRAIN Laboratory, Yaoundé, Cameroon, until shipping on dry ice a few weeks later. The frozen blackfly samples were then sent to the Laboratory of Viral Metagenomics in Leuven, Belgium, where they were stored at −80°C until the nucleic acids were extracted for sequencing.

### Sample processing

A total of 55 pools of 10 blackflies were analysed. The NetoVIR protocol (Conceição-Neto et al. 2015) was adapted for the homogenization of the blackfly pools. Homogenization was carried out in 2 mL tubes with 2.8 mm zirconium oxide beads at 5000 rpm for 15 seconds. Negative controls were added in the beginning and taken through the whole process (including sequencing) to make sure no contamination happened during the processing of the samples. Samples were centrifuged and filtered through a 0.8 µm filter (Sartorius) to remove cellular debris and enrich the samples for viral particles. This filtrate was treated with a mix of Benzonase (50 U, Novagen) and micrococcal nuclease (2000 U, New England Biolabs) to digest free-floating nucleic acids. DNA and RNA were simultaneously extracted using the QiaAMP Viral RNA Mini kit (Qiagen) without carrier RNA. A random amplification of both DNA and RNA with the Whole Transcriptome Amplification (WTA2) kit increased the nucleic acid concentration of the samples, and resulting PCR products were further purified and prepared for sequencing with the Nextera XT kit (Illumina). The final sequencing libraries were cleaned up with Agencourt AMPure XP beads (Beckman Coulter, Inc.) using a 0.6 ratio of beads to sample. Paired-end sequencing (2 × 150 bp) was performed on the Novaseq 6000 SP platform at the Nucleomics Core sequencing facility (VIB Leuven).

### Raw reads processing and virome analysis

To process the raw paired-end Illumina reads, an in-house bioinformatics pipeline (ViPER v1.1; https://github.com/Matthijnssenslab/ViPER) was used with the ‘triple assembly’ option enabled. Briefly, adapters and low-quality reads were trimmed with Trimmomatic (Bolger et al. 2014), before a *de novo* assembly with metaSPAdes v3.15.3 (Nurk et al. 2017). Resulting FASTA files with scaffolds were subsequently combined. To remove redundancy in the data, these scaffolds were clustered on 95% nucleotide identity over 85% coverage of the shortest sequence with the clustering algorithm available from CheckV (Nayfach et al. 2020). A length filter of 1000 nucleotides was applied on the sequences before viruses were identified in the data using a combined approach. This approach consisted of (i) genomad v1.7.0 on default setting with score calibration enabled (Camargo et al. 2024); (ii) DIAMOND blastx v2.0.11 on the sensitive setting (Buchfink et al. 2014) with NCBI’s nr database (accessed 23 March 2023); and (iii) a Hidden Markov Model (HMM) search of the scaffolds’ open reading frames, predicted by prodigal (Hyatt et al. 2010), against the NeoRdRP HMM dataset (v1.0) (Sakaguchi et al. 2022) with HHMER v3.3.2 (Eddy 2011) with a minimum *e*-value of 1*e*−10. Taxonomy assignment for blastx-identified sequences was decided based on a lowest common ancestor approach from the best 25 hits. When possible, the geNomad taxonomy output was prioritized for a sequence over the blastx taxonomic assignment. Subsequently, for quality control purposes, CheckV v1.0.1 (Nayfach et al. 2020) was employed to assess the genome completeness of the identified viral sequences. Sequences not belonging to *Riboviria* and less than 50% complete as predicted by CheckV were removed from further analysis. *Riboviria* sequences predicted to be less than 50% complete were kept because CheckV does not handle (segmented) RNA viruses well, which often results in wrong completeness estimations for RNA viruses. Finally, using the sequencing data of the negative controls, sequences that were predicted as contaminants by the prevalence method of decontam v1.22.0 (Davis et al. 2018) were removed from our dataset.

### Phylogenetic analysis

Based on the taxonomic assignment of the scaffolds (see above), we downloaded the International Committee on Taxonomy of Viruses (ICTV) representative protein sequences of each family or order present in our dataset (from ICTV’s MSL39 v1 VMR) with NCBI Virus. Except for the Orthomyxoviridae, of which representative PB1 sequences were downloaded from https://github.com/evogytis/orthomyxo-metagenomics/blob/main/data/Fig.3/PB1_full.fasta (Dudas and Batson 2023). For the RNA viruses, we first filtered out all non-RNA-dependent RNA polymerase (RdRP) proteins and partial RdRPs with a combination of palm_annot (https://github.com/rcedgar/palm_annot; commit 15d9443) and palmscan2 (https://github.com/rcedgar/palmscan) to retrieve the intact palmcore domain (Babaian and Edgar 2022). For the Genomoviridae and Parvoviridae, we downloaded the Rep and NS1 protein sequences, respectively. For each family or order, a diversified ensemble was created with Muscle5 (Edgar 2022). This diversified ensemble constitutes 100 multiple sequence alignments (MSAs) of the sequences with perturbed alignment parameters and permuted guide trees (see Muscle5 documentation for more information). From these 100 MSAs, the alignment with the highest column confidence, calculated with muscle maxcc, was picked to generate the final phylogenetic tree. Resampling of the diversified ensemble to generate support values for the phylogenetic tree was obtained with muscle resample -conf 0 -gapfract 0.5. Maximum likelihood (ML) phylogenetic trees from the resampled MSAs were inferred with FastTree (Price et al. 2010) (-lg -gamma -nosupport). These ML trees served as support for the tree that was generated from the maxcc MSA (also generated with FastTree and unaltered settings), which was calculated with newick conftree (https://github.com/rcedgar/newick).

### Viral host inference

To infer if the *Flaviviridae* and *Rhabdoviridae* viruses in our data could have an invertebrate or vertebrate host, we calculated the relative dinucleotide abundance (RDA) of our sequences and sequences with a known host (see Supplemental Table S2 for accession numbers) with DinuQ (Lytras and Hughes 2020). Next, we performed a principal component analysis in R on all dinucleotide RDA values and plotted the first against the second principal component for all viruses.

### Ethical approval

Approval was obtained from the ethics committee of the Cameroon Baptist Convention Health Services (reference number IRB2021-03). We also obtained a research permit from the Ministry of Scientific Research and Innovation (Ref.: 000144/MINRESI/B00/C00/C10/C13), as well as an ABS-Nagoya Protocol Prior Informed Consent (Ref.: Decision No. 00016/D/MINEPDED/CNA) and ABS Permit No. 00013/MINEPDED/CAN/NP-ABS/ABS-FP) from the Ministry of Environment, Protection of Nature and Sustainable Development.

## Results

### Identification of more than thousand viral contigs

For the 55 blackfly pools sequenced, between 6 113 870 and 24 002 232 reads were obtained per pool. In total, around 720 million raw reads were generated from all 55 pools. Raw read classification with Kraken2 (Wood et al. 2019) showed that most reads could not be directly classified, followed by on average 30% of the reads assigned to bacteria (see Supplemental Fig. 1B). In addition, we did not find any nematode genome sequences, while in most samples fungal sequences were abundantly present (see Supplemental Fig. 2). After trimming, each pool retained between 4 544 534 and 20 790 370 reads, resulting in a 17.5% loss of the initial raw reads (see Supplemental Table S1 and Supplemental Fig. 1A). Subsequently, *de novo* assemblies were generated, resulting in a total of 59  645 scaffolds larger than 1000 bp from the 55 pools. The clustering of the assembled scaffolds from all samples based on 95% nucleotide identity over 85% coverage resulted in 45 308 non-redundant scaffolds. After removing contamination predicted with decontam, a total of 1678 scaffolds were identified as viral with a combination of geNomad, DIAMOND blastx, and an HMM search against the NeoRdRP dataset (see Supplemental Fig. 3A). Of these, 56 were considered to be 100% complete, 128 were of high quality (>90% complete), and 190 of medium quality (>50% complete); the remaining viral sequences were either of low quality or their completeness could not be determined by CheckV (see Supplemental Fig. 3B). However, the low-quality and not-determined groups consisted only of sequences with *Riboviria* or unclassified taxonomic realm assignments. This suggests that these sequences are significantly divergent and might thus be of a higher quality than what is estimated by CheckV (also see Materials). Overall, we concluded that, due to the high convergence of our three identification methods and the completeness estimation of CheckV, we have a robust set of identified viruses.

### The blackfly virome diversity is dominated by RNA viruses

**Figure 1.**
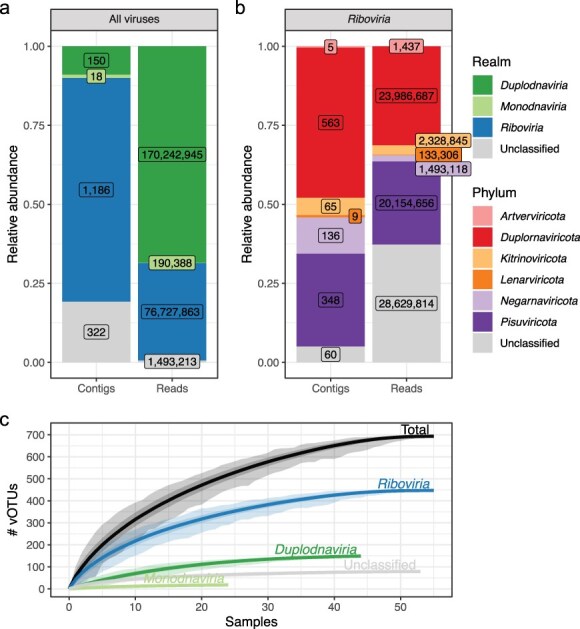
Blackfly virome diversity is dominated by RNA viruses. (a) Barplots showing the relative abundance of all identified viruses at contig and read level. Labels indicate the absolute numbers. (b) Barplots showing the relative abundance of all identified *Riboviria* phyla at contig and read level. Labels indicate the absolute numbers. (c) Rarefaction curves for each virus kingdom and the total. Light shaded areas indicate the minimum and maximum observed species across all iterations, the dark shaded areas indicate the 95% confidence around the mean. Virus operational taxonomic unitswere defined as sequences that clustered separately with our clustering parameters (see ‘Methods’ section) and contained an RdRP gene for the *Riboviria* or were >50% complete for the other taxa.

When examining the taxonomic distribution of our viral genome sequences, the majority (n = 1186) were classified within the *Riboviria* realm, 322 could not be assigned into a specific realm, and a small number belonged to *Monodnaviria* and *Duplodnaviria* (see Fig. 1A). At the read level, a distinct picture arises with approximately two-thirds being identified as belonging to the *Duplodnaviria* realm. This is mostly because the average viral genome length of *Duplodnaviria* is much higher than that of *Riboviria*, which in our data recruit about one-third of the viral reads (see Fig. 1A and Supplemental Fig. 4). The 322 unclassified sequences recruit only a small fraction (∼1.5 million) of the reads. As the diversity seemed the greatest in the *Riboviria* realm, which also contains the vast majority of arboviruses, we further investigated the taxonomic distribution of their scaffolds as well as their reads. Around half of the viral genome sequences belonged to the *Duplornaviricota* phylum (see Fig. 1B), while this phylum constituted only one-third of the *Riboviria* reads (see Fig. 1B). Surprisingly, 81 further unclassified *Riboviria* sequences were responsible for another one-third of the viral reads in our blackfly samples. These results show that the blackflies contained a high number of completely novel RNA viruses that were present in high abundance. Moreover, we constructed rarefaction curves to assess the depth of sampling required to encompass the full spectrum of viral diversity (Fig. 1C). Notably, the curves (total and *Riboviria*) plateaued after approximately 50 pools, suggesting saturation and the absence of novel viral taxa beyond this point. These findings indicate that our sampling efforts comprehensively cover the viral diversity within the blackflies from the onchocerciasis-endemic Nachtigal village (Cameroon).

**Figure 2.**
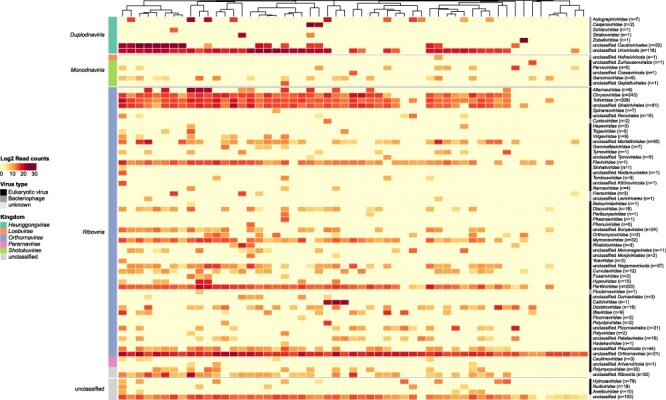
Blackflies harbour a large number of viral families, including families of potential clinical interest. Heatmap of all viral families per blackfly pool based on the blastx results (sequence similarity cutoff of at least 70% was used to assign scaffolds to families, otherwise they were assigned to closest related order). The heatmap shows the read count on a log_2_ scale for eukaryotic virus on family level from all the 55 adult blackfly pools. The number of identified scaffolds is indicated after each viral family name in the heatmap. Hierarchical clustering of the columns is based on the Bray–Curtis distance calculated from the read counts.

To visualize the virome similarity of the 55 pools, a heatmap with the read count for each viral family on a log_2_ scale was constructed (see Fig. 2). In total, 40 eukaryotic viral families were identified across all samples. In addition, 11 different taxonomic instances of prokaryotic viruses were identified on the family level. Most diversity in the blackflies on the family level is present in the *Riboviria* realm with 35 families of eukaryotic RNA viruses detected.

The viral families *Flaviviridae*, *Hepeviridae*, *Orthomyxoviridae*, *Parvoviridae*, *Phenuiviridae*, *Spinareoviridae*, *Rhabdoviridae*, and *Togaviridae* include human pathogenic viruses, some of which may also be arboviruses. Notably, known viruses causing neurological complications belong to the families of *Rhabdoviridae*, *Flaviviridae*, and *Picornaviridae*, as well as *Togaviridae*.

Fungal viruses were the most prevalent viruses, with members of *Ghabrivirales* (i.e. *Chrysoviridae*, *Totiviridae*) and *Partitiviridae* being the most abundant. A virus of the family *Flaviviridae* was present in most blackfly pools and in a relatively high abundance. Furthermore, other eukaryotic viral families (main hosts according to ICTV are given in brackets) that were prevalent were *Curvulaviridae* (fungi), *Dicistroviridae* (invertebrates), *Iflaviridae* (insects), *Genomoviridae* (humans, mammals, birds, fungi), *Mymonaviridae* (fungi), *Orthomyxoviridae* (humans, birds, pigs), *Parvoviridae* (vertebrates, insects), *Rhabdoviridae* (vertebrates, invertebrates, plants), *Totiviridae* (fungi), and *Virgaviridae* (plants). From these families we constructed phylogenetic trees, either grouped in their respective order or as a separate family.

### RNA virus phylogenetics

To fully display the diversity of RNA viruses in the blackflies, we made phylogenetic trees of the palmcore sequences of the RdRp protein for the most common and most interesting RNA viruses present. These palmcore sequences might not completely reflect the true phylogeny of the viruses in the tree, but they prove to be a robust way to taxonomically classify sequences, as shown by the high monophylicity of established families and genera (see Supplemental Figs. 5–14).

**Figure 3.**
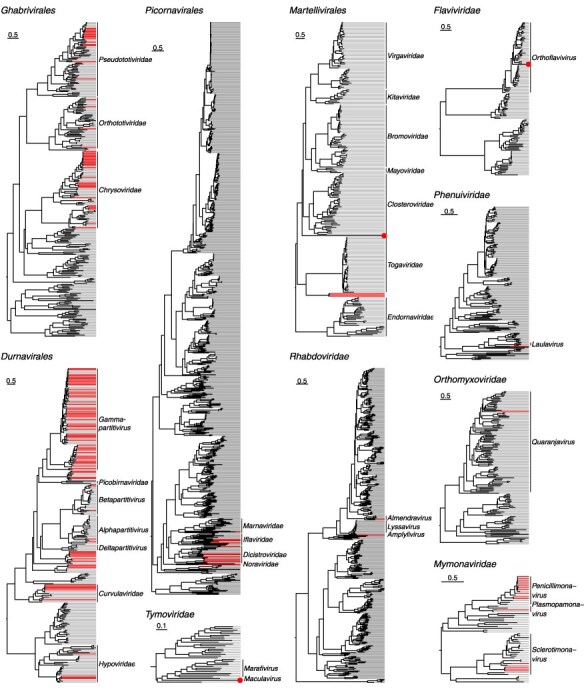
Phylogenetic trees representing the major RNA virus orders and families identified in the blackflies. Coloured lines signify novel viruses identified in the blackflies, while grey lines are ICTV references. The scale bars indicate the number of amino acid substitutions per site. Families and/or genera closest to the novel viruses are shown at the tips of the tree with a bar covering all ICTV exemplar species within that taxonomy (or no bar when there is only one exemplar species) .

The majority of the viruses in the blackflies seem fungi-infecting as they are part of the *Ghabrivirales*, *Durnavirales* (dsRNA) and *Mymonaviridae* (ssRNA−) and cluster together with ICTV exemplar viruses that infect fungi (see Fig. 3 and Supplemental Figs. 5–7, respectively). Within the dsRNA orders, the blackfly virus sequences often form deep branches in the tree, suggestive for the discovery of new species, genera or maybe even families, thereby expanding the known diversity of the *Partitiviridae* and *Curvulaviridae* in the *Durnavirales*, and the *Chrysoviridae* and *Pseudototiviridae* within the *Ghabrivirales*.

Viruses truly infecting the blackflies are mostly found in the *Picornavirales* order (ssRNA+), within the *Iflaviridae* and *Dicistroviridae* (see Fig. 3 and Supplemental Fig. 8). Furthermore, within *Martellivirales*, which contains mostly plant-infecting viruses apart from *Togaviridae*, we have possibly two new families. Interestingly, the viruses most closely related to the *Togaviridae* members are probably segmented viruses as the recovered sequences only contain an RdRP gene. Attempts to retrieve more segments with a contig co-occurrence analysis (Batson et al. 2021) were unfortunately unsuccessful due to the low prevalence of these viruses in the blackfly samples. Also, blastx searches against NCBI’s nr database revealed only sequences with only an RdRP gene and without a matching segment in NCBI’s nt database coding for a structural protein/proteins.

Furthermore, we have a few singular viruses in the families *Tymoviridae*, *Flaviviridae*, *Phenuiviridae*, and *Orthomyxoviridae*, of which the host is most likely the blackfly. In the *Rhabdoviridae*, we find two viruses: one that is clearly a member of the *Almendravirus* genus, which contains invertebrate-infecting viruses, and one virus that is not a part of a known genus but is part of a large clade that contains viruses infecting vertebrates and invertebrates as well as both (see Supplemental Fig. 14).

### Novel blackfly rhabdo- and flaviviruses are invertebrate-specific

**Figure 4.**
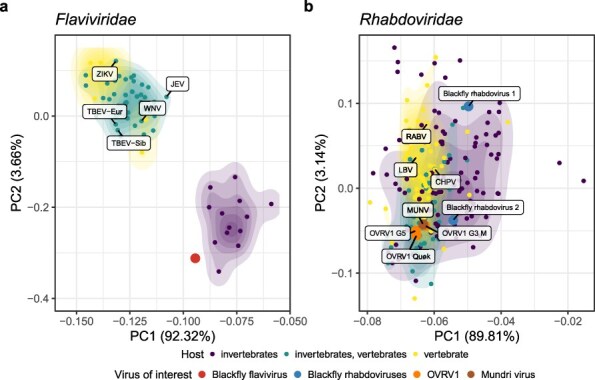
Identified blackfly flavi- and rhabdoviruses are insect-specific. (a) Principal component analysis (PCA) plot based on the RDA of selected *Flaviviridae* member sequences. The largest dot shows the newly identified flavivirus in blackflies. Labels show a selection of neurotropic flaviviruses (TBEV = tick-borne encephalitis virus, JEV = Japanese encephalitis virus, WNV = West Nile virus, ZIKV = Zika virus). (b) PCA plot based on the RDA of selected *Rhabdoviridae* member sequences. The large blue dots show the newly identified blackfly rhabdoviruses, the large orange dots show different variants of OVRV1, the large brown dot shows MUNV. Other labelled dots show a selection of neurotropic rhabdoviruses (CHPV = Chandipura virus, LBV = Lagos bat virus, RABV = rabies virus) .

To investigate whether the *Rhabdoviridae* and *Flaviviridae* member viruses we found in the blackflies could also infect vertebrates, we compared the dinucleotide composition within their genomes with the genomes of viruses with a known host. After colouring the viruses by host, this revealed a clear clustering in vertebrate-infecting vs. insect-specific viruses for the *Flaviviridae* (see Fig. 4A), meaning that this family of viruses adapted itself to avoid the vertebrate immune system by using similar dinucleotide abundances in their genome (Lytras and Hughes 2020, Fros et al. 2021). For the *Rhabdoviridae*, this clustering is less clear, but there seems to be nevertheless a small difference. The flavivirus we found in the blackflies shows a dinucleotide profile suggestive of being an insect-specific virus, and also the rhabdoviruses we found in the blackflies seem to be infecting invertebrates when combining both the RDA information and the phylogenetic analysis (see Fig. 4B and Supplemental Fig. 14). Additionally, we added two other virus species recently identified in other studies to the *Rhabdoviridae* dinucleotide analysis: Onchocerca volvulus RNA virus 1 (OVRV1) is a rhabdovirus that was found in *O. volvulus* and elicits an immune response in the host (human as well as cattle and hamsters) (Quek et al. 2024), while MUNV was detected in a patient with nodding syndrome (Edridge et al. 2022). Interestingly, both multiple OVRV1 variants as well as MUNV seem to cluster with vertebrate-infecting rhabdoviruses, suggesting that these viruses may be capable of infecting vertebrates.

## Discussion

To the best of our knowledge, this study represents the first comprehensive investigation of DNA and RNA viruses in blackflies utilizing a viral particle-based whole-genome sequencing approach. A very high number of virus genomes, with a dominance of RNA viruses, were identified in blackfly samples. Fifteen good quality (>50% complete) genomes of eukaryotic DNA viruses (e.g. belonging to the *Parvoviridae* and *Genomoviridae*) and 641 RNA viruses (based on the RdRP, of which 466 with a complete palmprint) were detected. A significant proportion of the novel viruses belonged to viral families associated with human disease, e.g. *Flaviviridae*, *Hepeviridae*, *Orthomyxoviridae*, *Parvoviridae*, *Phenuiviridae*, *Picornaviridae*, *Spinareoviridae*, *Rhabdoviridae*, and *Togaviridae*. However, no viral genomes closely related to known neurotropic viruses were found in the blackfly virome (Supplementary files). Obviously, this does not mean that none of the newly identified viruses could be arboviruses with a neurotropic potential. Additionally, the real host origin for many of the newly discovered viruses remains unknown, and further investigations are necessary to determine whether these viruses have the potential to replicate in vertebrate and/or invertebrate hosts, or even if they have a prokaryotic host as could be the case for members of the *Partitiviridae* and *Picobirnaviridae* (Neri et al. 2022, Sadiq et al. 2024).

The only *Flaviviridae* virus in our analysis was observed at a relatively high abundance in most blackfly samples (see Fig. 2). Previous phylogenetic analyses and experimental studies (Kuno and Chang 2005, Cook et al. 2013) have shown that the phylogenetic tree of the *Flaviviridae* separates into two large clusters. The first cluster contains insect-specific viruses, which appear to be host-restricted and only replicate in invertebrate cells and primarily infect mosquitoes (Baidaliuk et al. 2020, Hoshino et al. 2009, Guzman et al. 2018, Miranda et al. 2019). The second cluster comprises human pathogenic arboviruses. These previous studies also indicate that arthropod-borne viruses of vertebrates may have originated from insect-specific viruses that evolved from being limited to insects to acquiring the ability to infect vertebrate hosts (Bolling et al. 2015). The dinucleotide analysis of the *Flaviviridae* indicated that the flavivirus in our blackfly samples is most likely an insect-specific virus.

As blackflies can act as intermediate hosts of pathogens affecting humans and animal health due to their haematophagic habit, several studies (Mead et al. 2004, Smith et al. 2009, Mesquita et al. 2017, Young et al. 2021) have focused on the transmission of vesicular stomatitis virus (VSV), an arbovirus belonging to the family *Rhabdoviridae*, genus *Vesiculovirus*, which is known to be transmitted by blackflies. This virus primarily infects livestock, representing an agriculturally relevant pathogen, but zoonotic events have also been reported, highlighting its potential impact on human health (Lichty et al. 2004). VSV infections are generally asymptomatic in humans; however, mild flu-like symptoms have been reported in some individuals, and a single case of encephalitis in a 3-year-old boy, which was potentially associated with VSV infection, has been reported (Quiroz et al. 1988).

Consistent with the findings of Edridge et al. (2022), our phylogenetic analysis of *Rhabdoviridae* members (see Supplemental Fig. 14) showed that MUNV is closely related to members of the *Tibrovirus* genus, supported by a high bootstrap value. However, the two sequences detected in the blackfly virome were not closely related to the *Tibrovirus* nor *Vesiculovirus* genera. In addition, another novel rhabdovirus, named OVRV1, was discovered by Quek *et al*. in public transcriptomes and viromes of *O. volvulus* nematodes (Quek et al. 2024). Because OVRV1 was shown to elicit an immune response in the vertebrate host of the nematode, it could potentially play a role in the pathogenesis of OAE (Quek et al. 2024). We were, however, not able to find this virus back in the virome of the blackflies. Our dinucleotide analysis for the *Rhabdoviridae* suggested that the viruses we found in the blackflies are invertebrate-specific viruses, in contrast to the previously identified rhabdoviruses MUNV (Edridge et al. 2022) and OVRV1 (Quek et al. 2024). However, a recent association between MUNV infection and nodding syndrome was investigated in a case–control study in South Sudan. MUNV RNA was only detected in the plasma of one of the 72 persons with nodding syndrome and in none of the controls. MUNV anti-nucleocapsid IgG antibodies were observed in 28% of nodding syndrome cases, 22% of household controls, and 16% of community controls without significant differences between cases and both control groups. Therefore, it was concluded that MUNV may not be causally associated with nodding syndrome (Edridge et al. 2022). Similarly, it remains to be shown whether OVRV1 may be associated with OAE.

Our study is the first to extensively study the DNA and RNA virome of blackflies from an onchocerciasis-endemic area in Africa. Our findings reveal a relatively high abundance of members of the family *Genomoviridae* in the majority of blackfly samples, aligning with the results of Kraberger *et* *al.*, which demonstrated that the majority of novel ssDNA viral genomes detected in blackflies in New Zealand belonged to the *Genomoviridae* family (Kraberger et al. 2019). However, in contrast with the blackflies in New Zealand (Kraberger et al. 2019), our study identified members of the *Parvoviridae* family in the blackfly virome. Although this viral family exhibited a high abundance, it was only observed in a limited number of blackfly samples. Phylogenetic analysis revealed that one of the *Parvoviridae* genomes detected in this study clustered within the *Densovirinae* subfamily (see Supplemental Fig. 16).

Most of the detected viruses exhibited low sequence similarities with their closest relatives. Consequently, based on the demarcation criteria established for viral classification, it is reasonable to propose that the majority of sequences identified in our study represent new virus species, genera, and/or families, which warrant further studies. In our project, plant and fungal infecting viruses (mainly members of the *Chrysoviridae* and *Partitiviridae* families) were the most abundant, possibly from the nectar diet of the blackflies. Some members of viral families of clinical interest were detected, albeit at lower abundances and no viral genomes closely related to neurotropic viruses were identified.

Our study has some limitations. First of all, it reports the composition of the blackfly virome, including both DNA and RNA viruses but without metadata that would have allowed further associations to be made with *O. volvulus* and potential environmental factors. Further research should compare the virome of blackflies with and without *O. volvulus* infection, and the sex and species of the blackflies should be taken into consideration. It is important to note that only a few blackfly species consume a blood meal (Kraberger et al. 2019). Additionally, as less than 1% of blackflies in Cameroon are currently infected with *O. volvulus* (Shintouo et al. 2020), it is recommended to screen blackflies for *O. volvulus* infection before sequencing data analyses, as previously suggested (Ekangouo et al. 2022).

## Conclusion

Overall, we provide a glimpse into the viral diversity associated with blackfly vectors in Cameroon. Although no genomes associated with neurotropic viruses were found, a plethora of new virus species, genera, and families were identified. The presence of dominant and under-represented viruses warrants future research to determine the role of these viruses in their hosts and ecosystems. Exploring the diversity of viruses in blackflies should be included in the active surveillance of zoonotic diseases. Our findings constitute a starting point for investigating the viruses associated with the haematophagous blackfly and potentially in their nematode host *O. volvulus*.

## Supplementary Material

veaf024_Supp

## Data Availability

Sequencing data are available in the SRA database under BioProject accession number PRJNA1088476. Identified viral genomes can be found in the following Zenodo repository with a corresponding taxonomy table: https://doi.org/10.5281/zenodo.13737160. In addition, all coding-complete viral sequences have been made available on GenBank (accession numbers PV236217–PV237137). The code used for this study is available in a GitHub repository: https://github.com/Matthijnssenslab/2025_VirusEvolution_BlackflyVirome.
