# Supplementary material for: Cameroonian blackflies (Diptera: Simuliidae) harbour a plethora of RNA viruses
Source: Virus Evol. 2025 Apr 5;11(1):veaf024. doi: 10.1093/ve/veaf024 (PMC12048880; doi:10.1093/ve/veaf024)
Supplement: veaf024_Supp [file veaf024_supp.zip › suppl_data/Virus_Evolution_Supplementary_Figures_and _Tables.pdf]

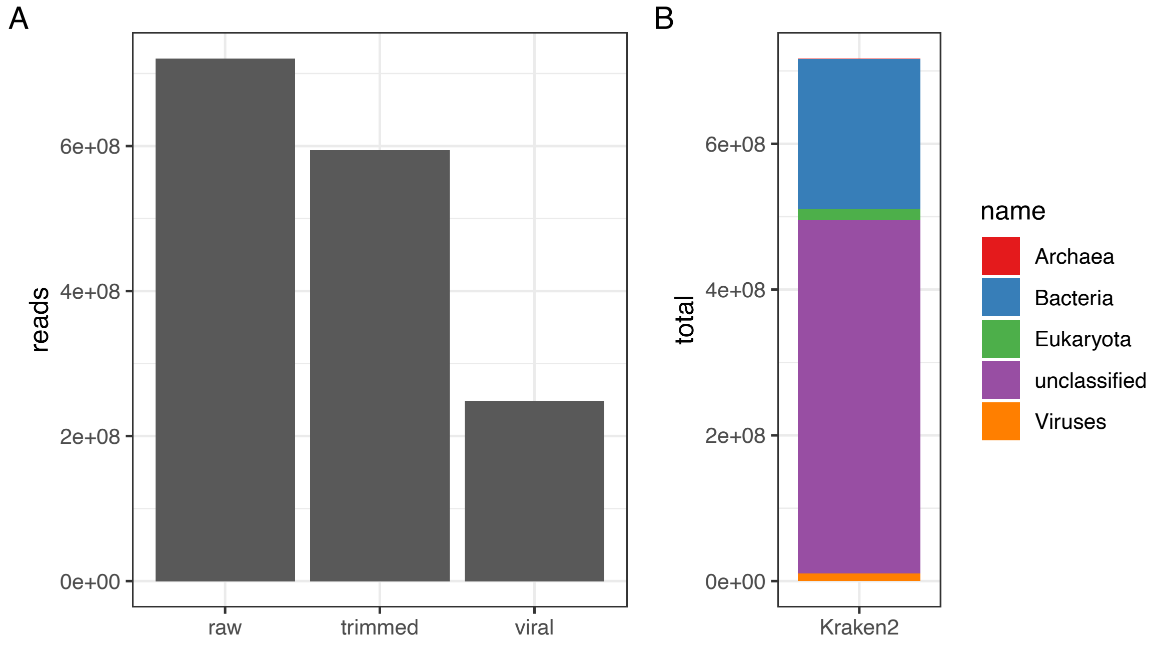

Supplementary Figure 1

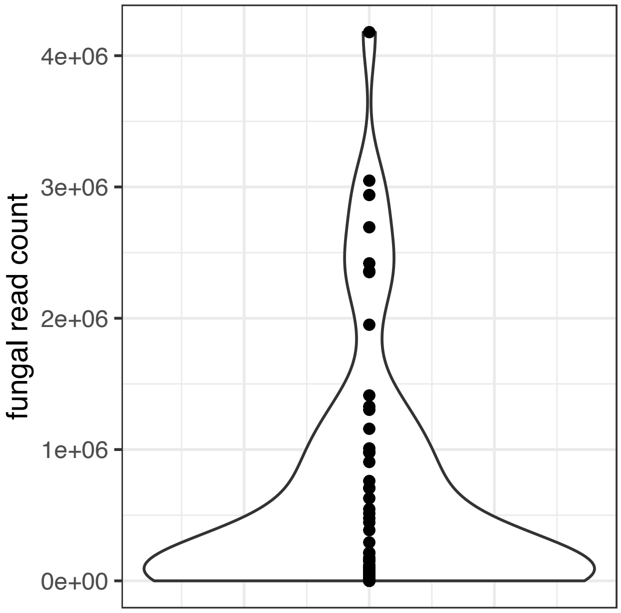

Supplementary Figure 2

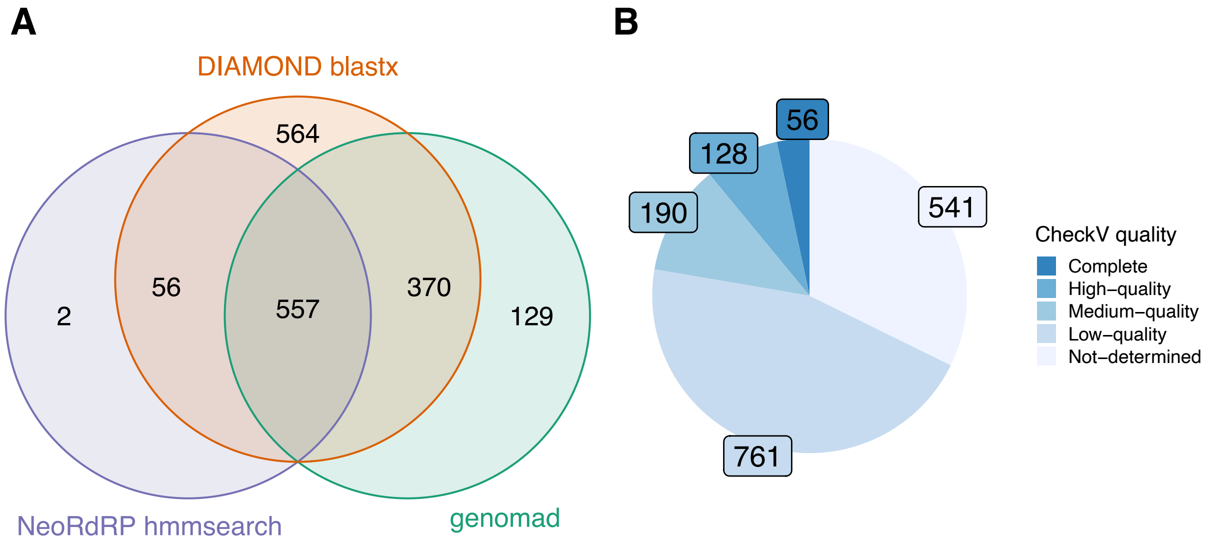

Supplementary Figure 3

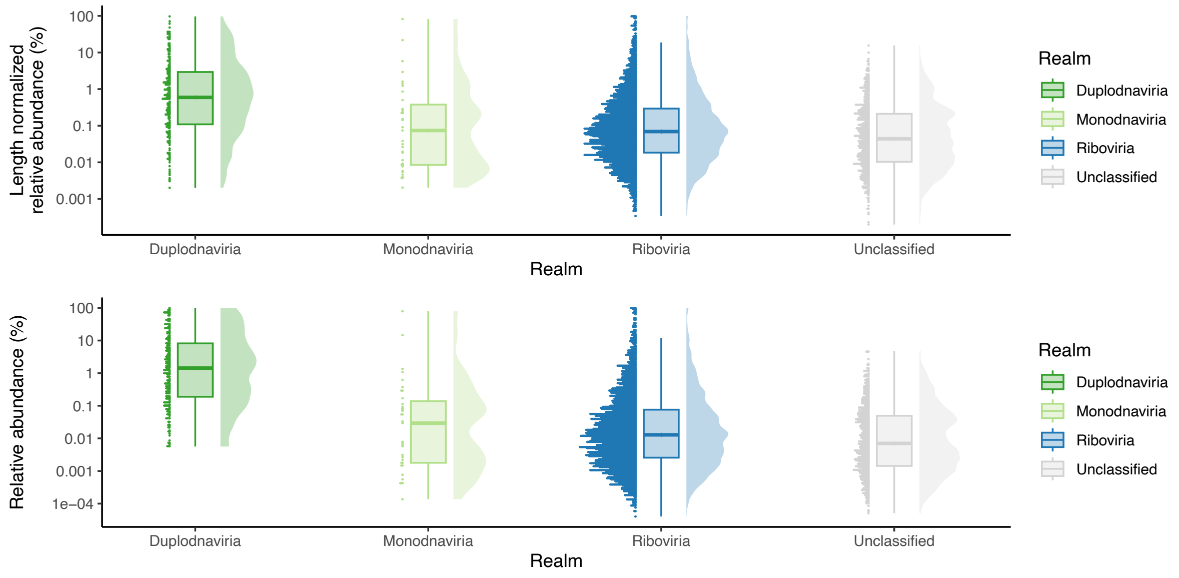

Supplementary Figure 4

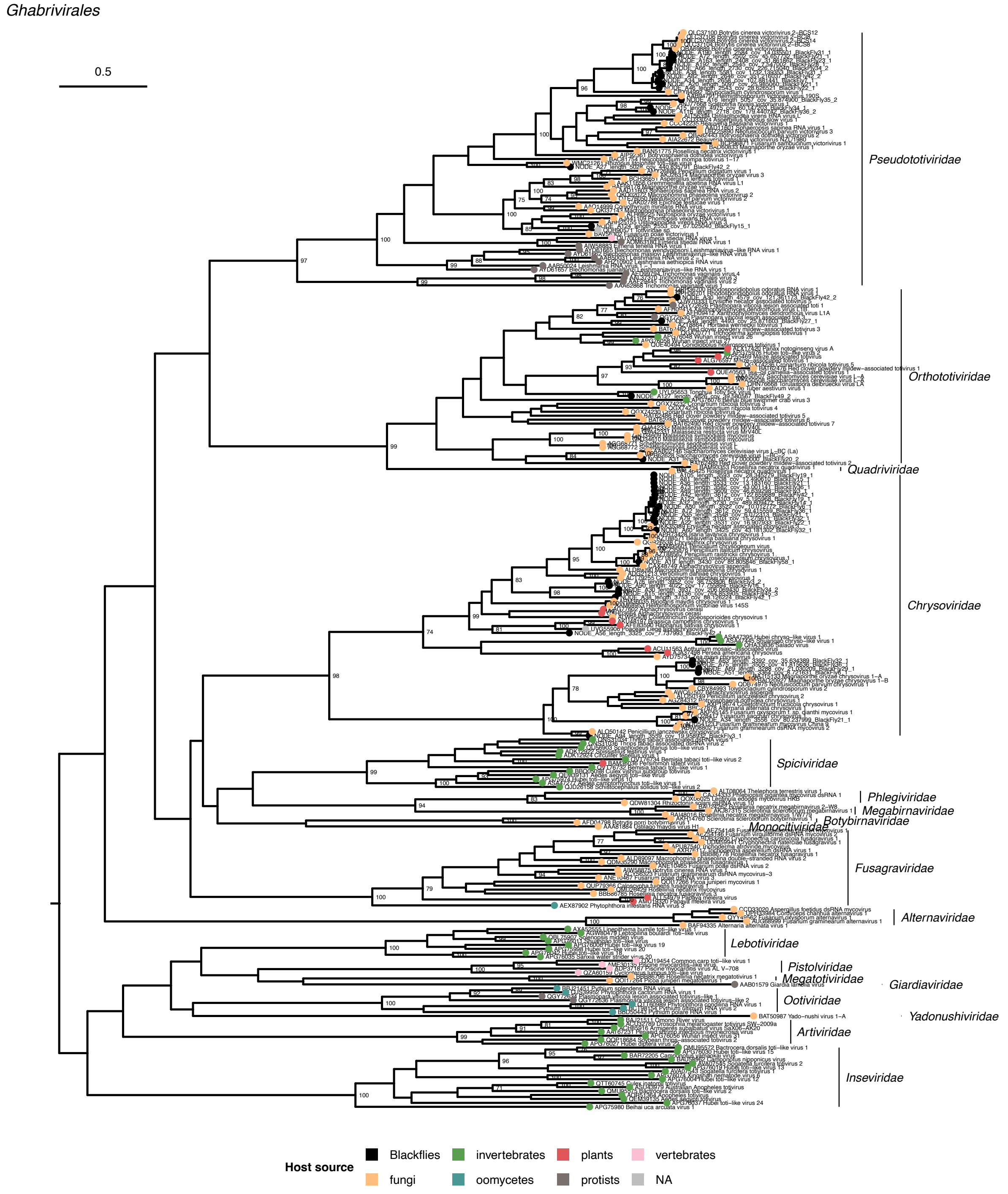

Supplementary Figure 5

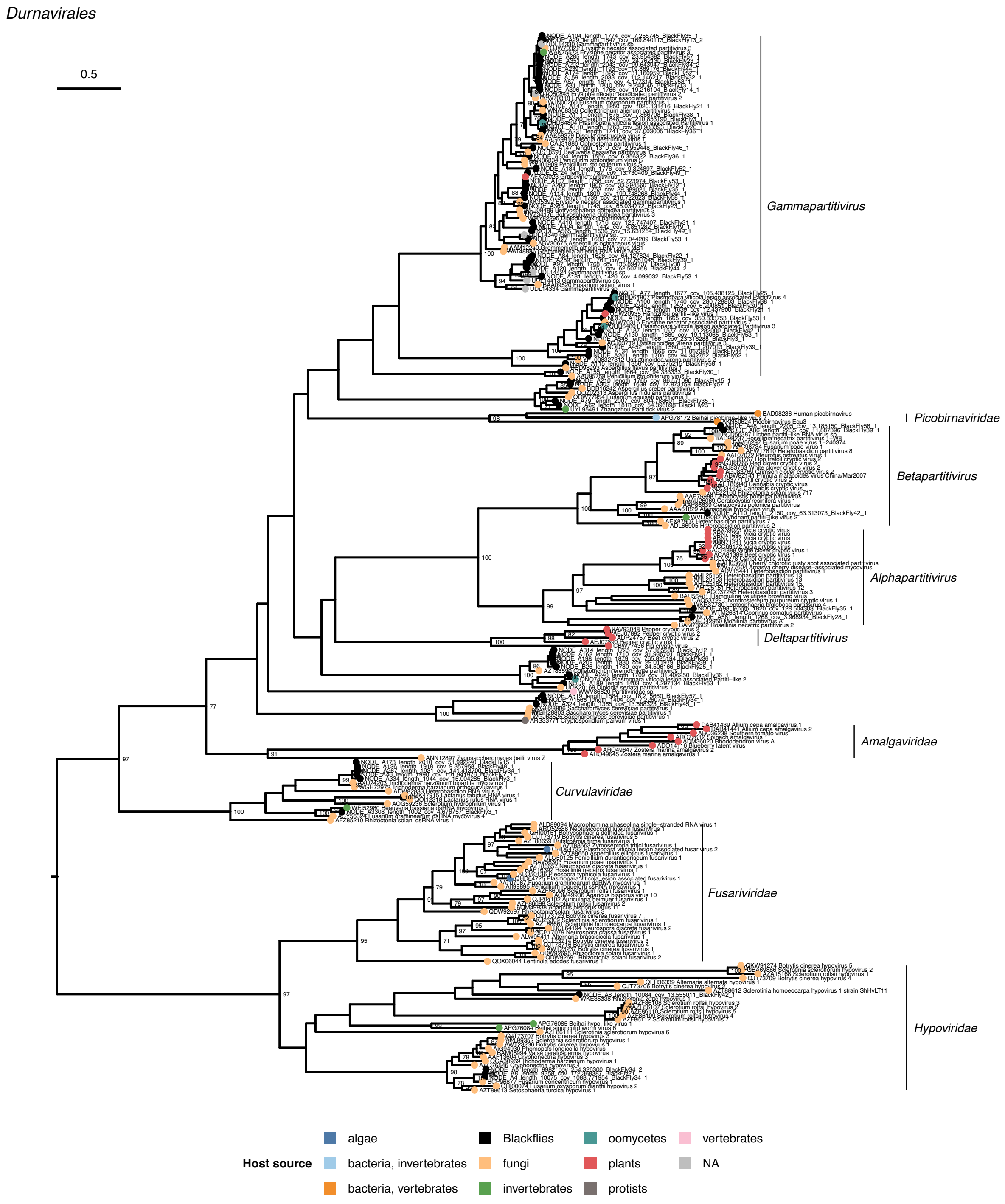

Supplementary Figure 6

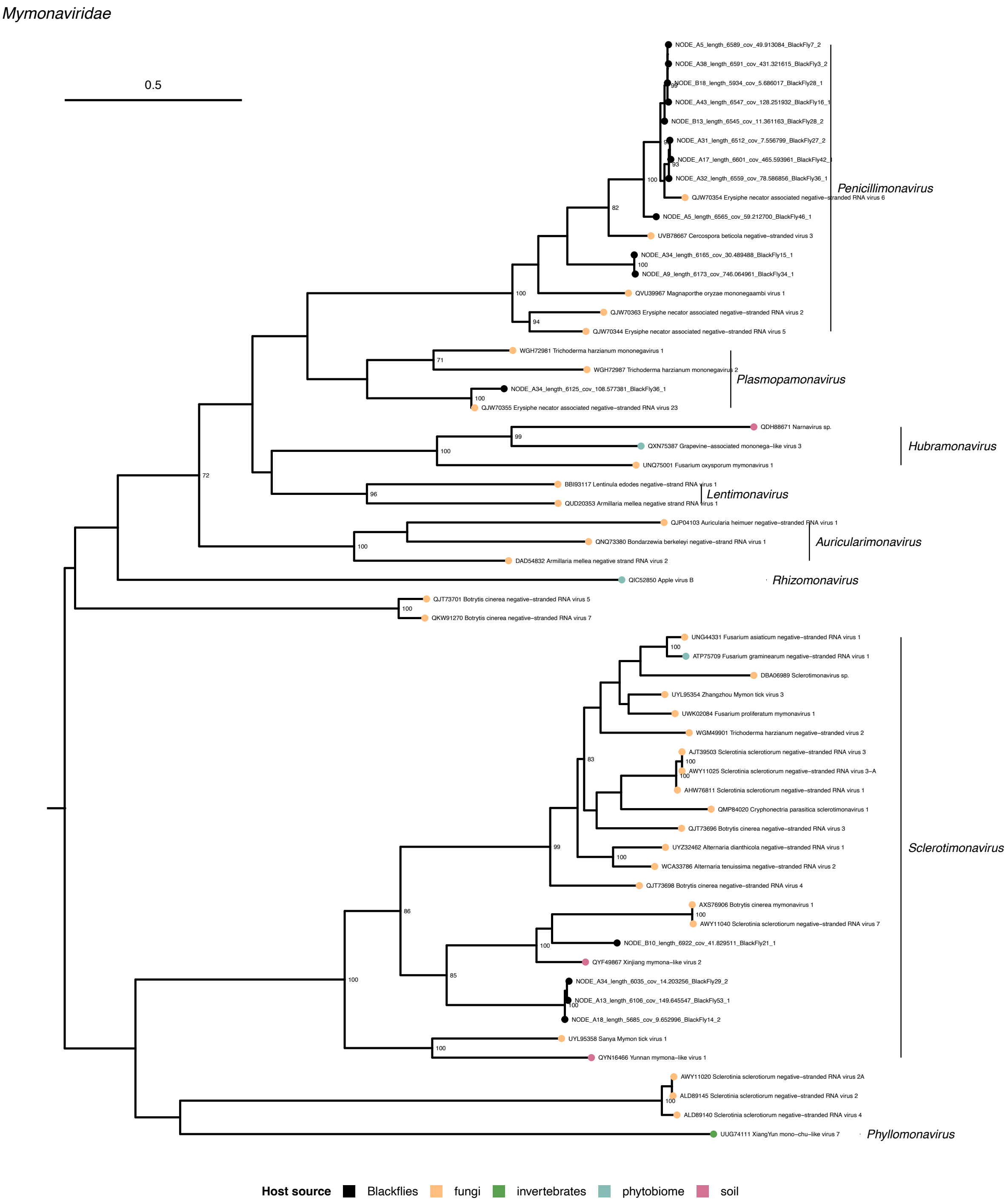

Supplementary Figure 7

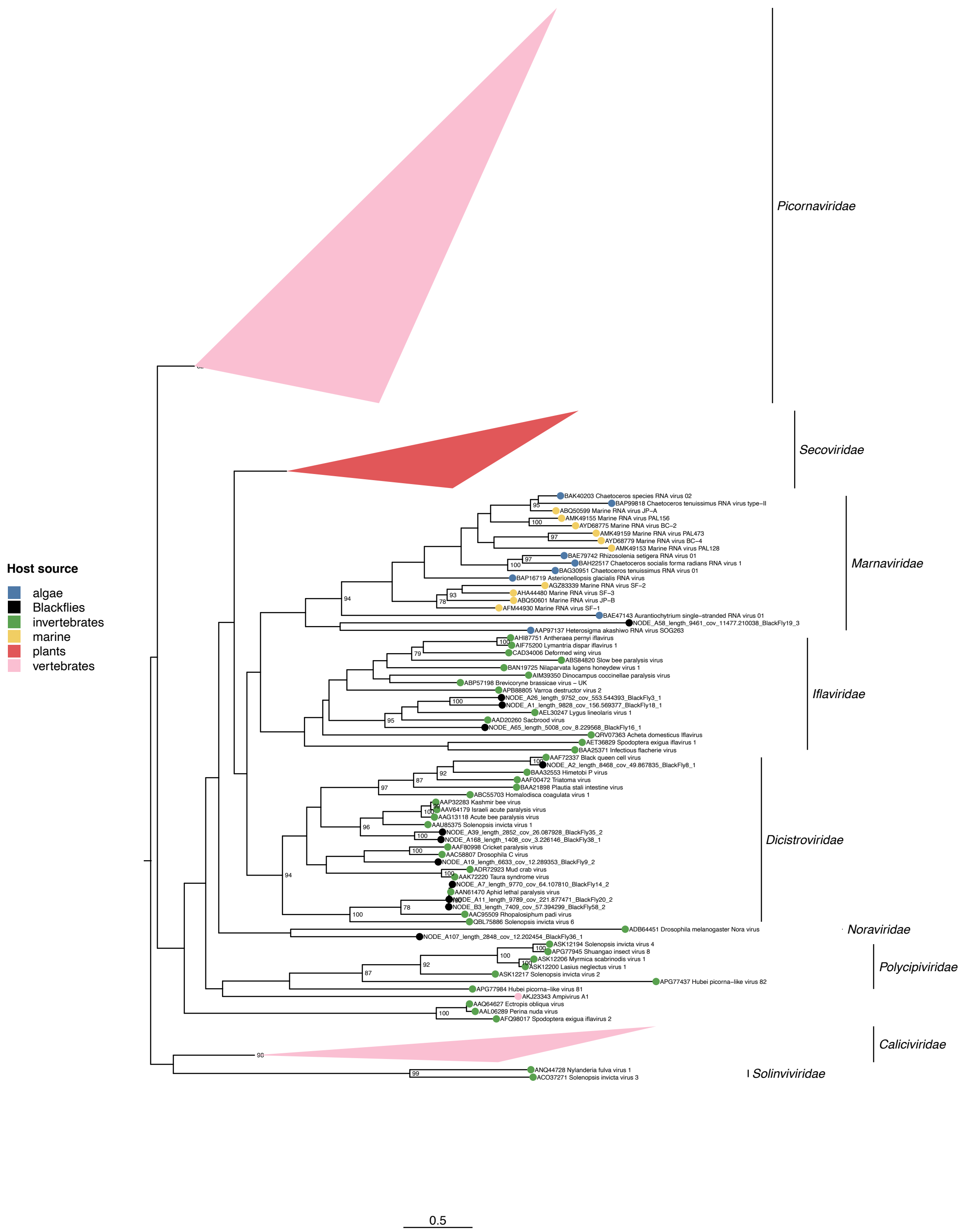

Supplementary Figure 8

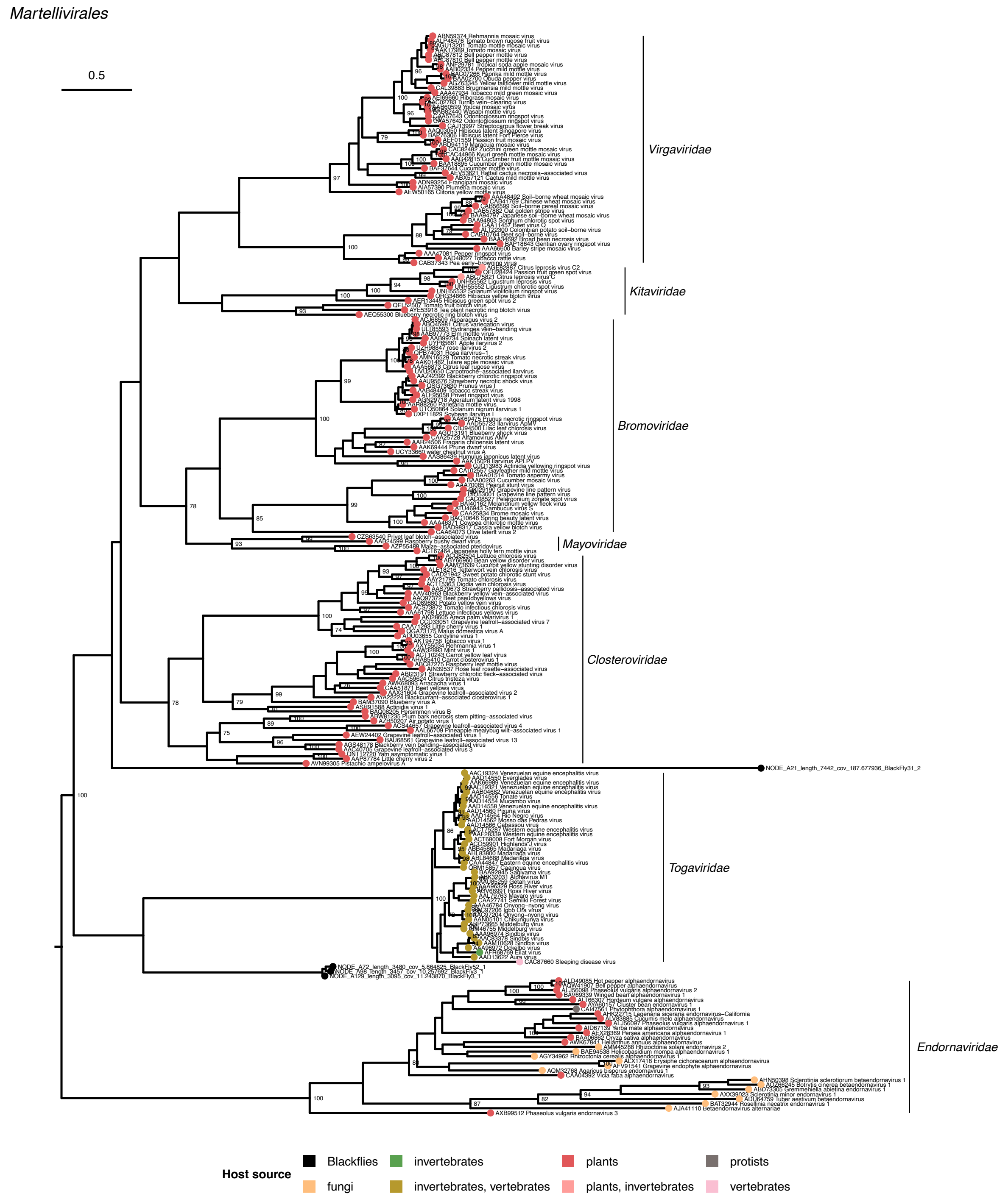

Supplementary Figure 9

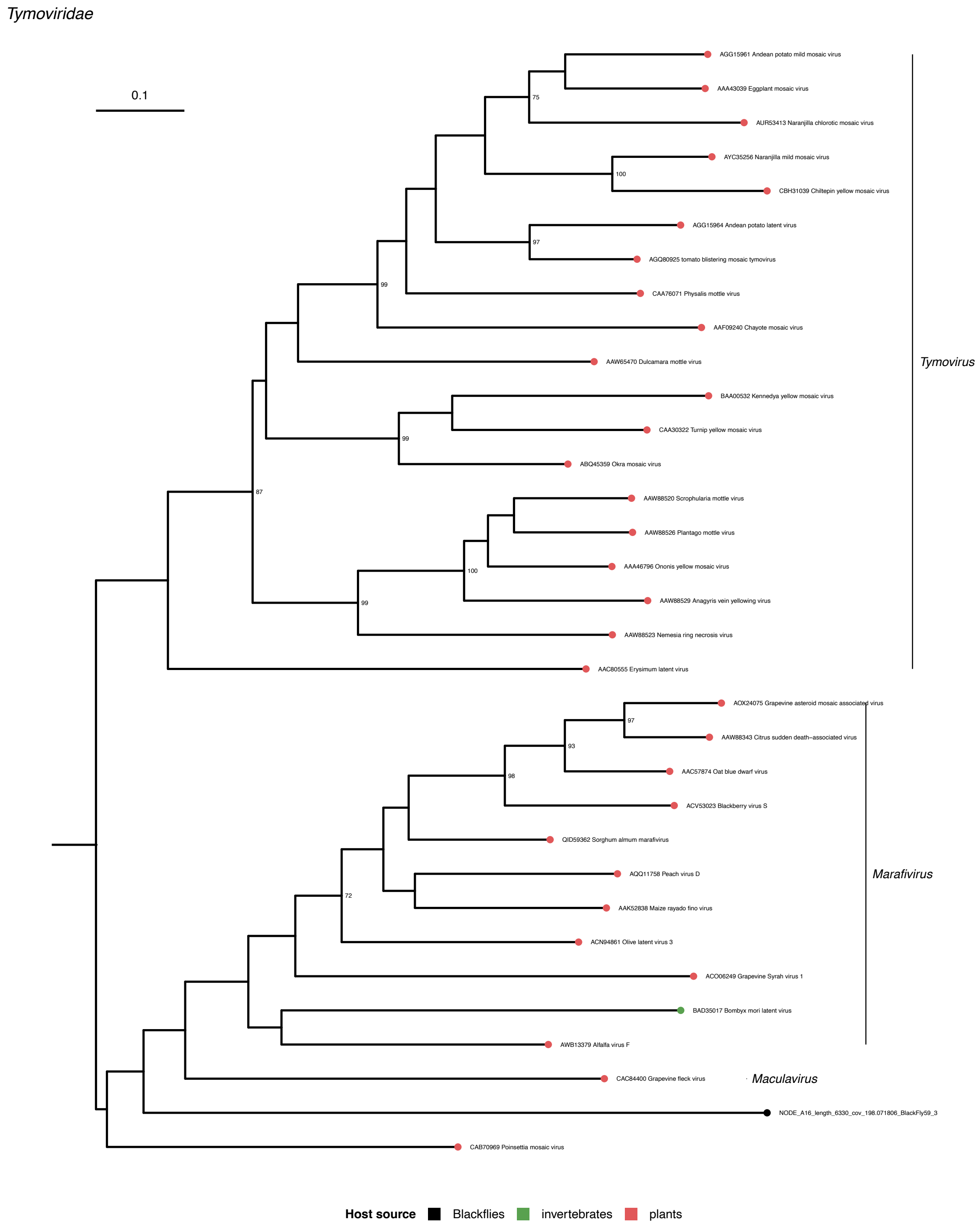

Supplementary Figure 10

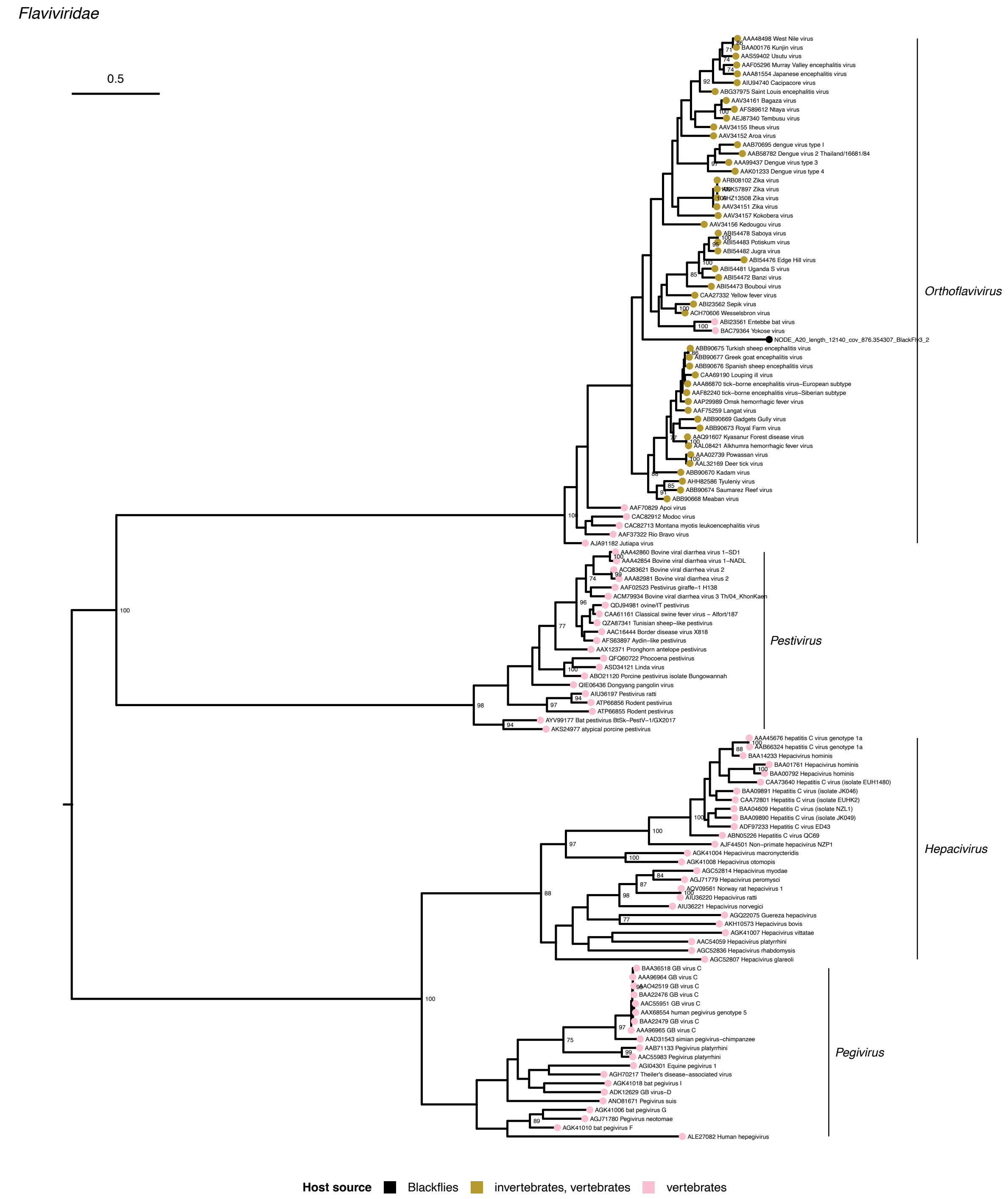

Supplementary Figure 11

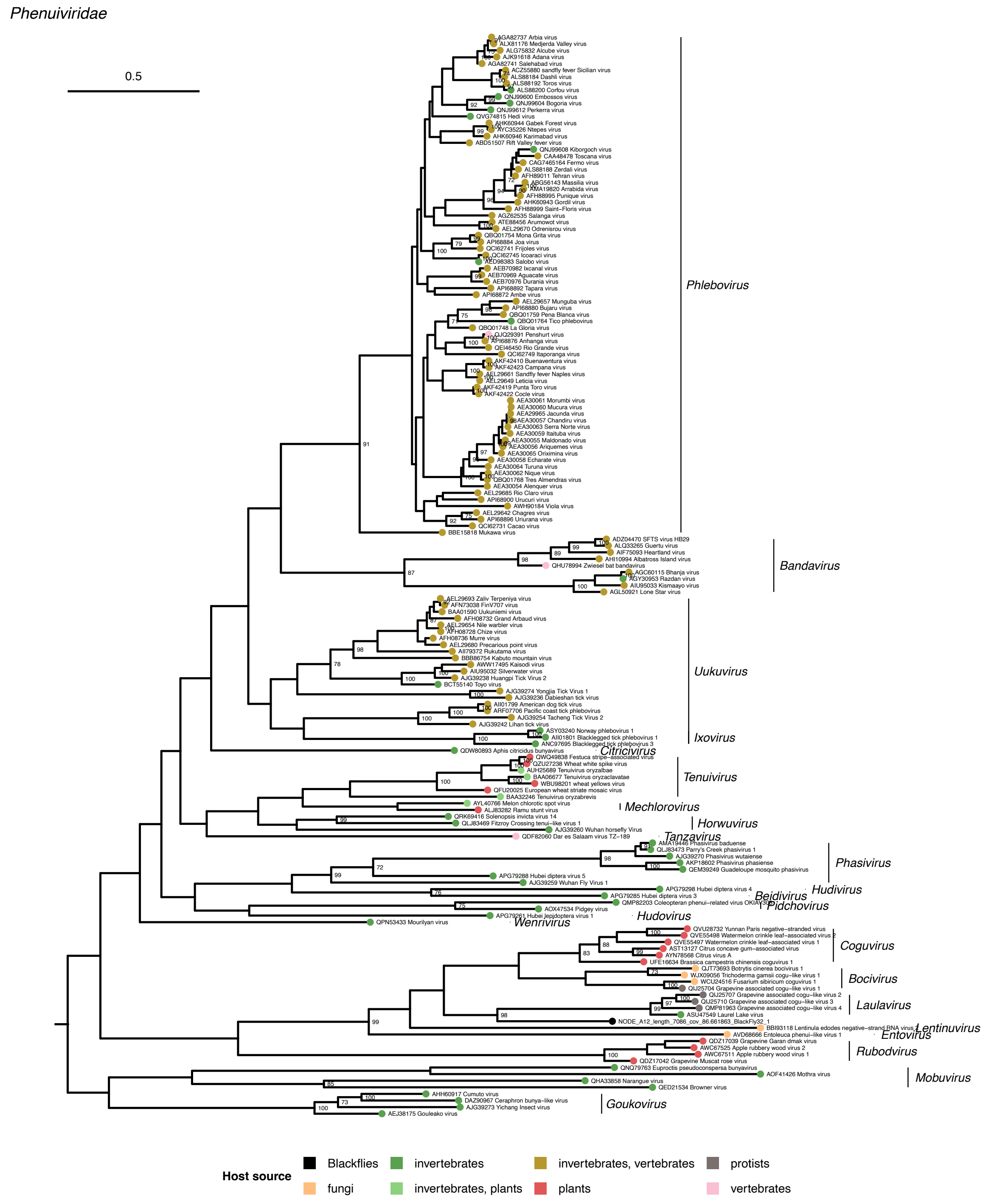

Supplementary Figure 12

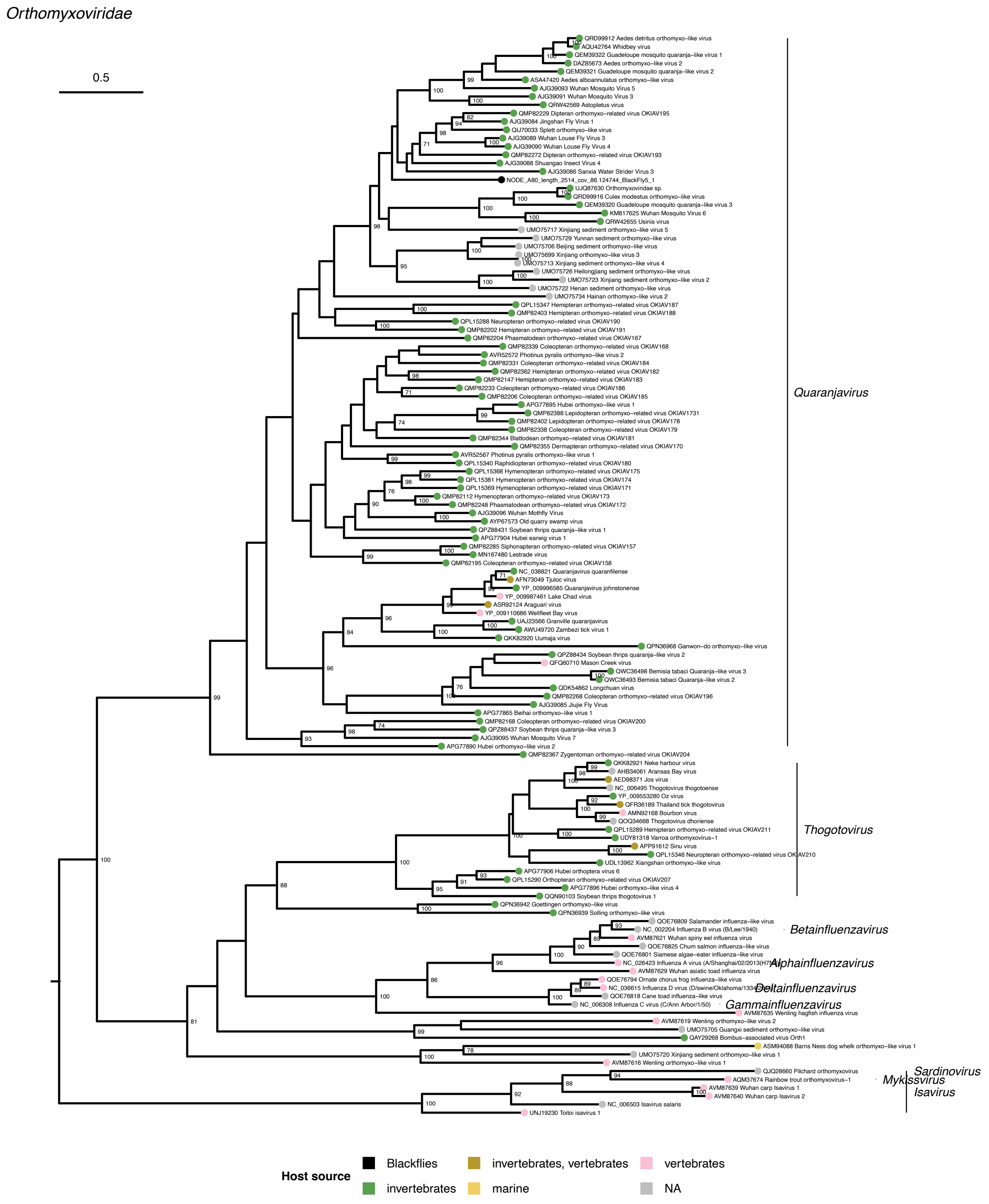

Supplementary Figure 13

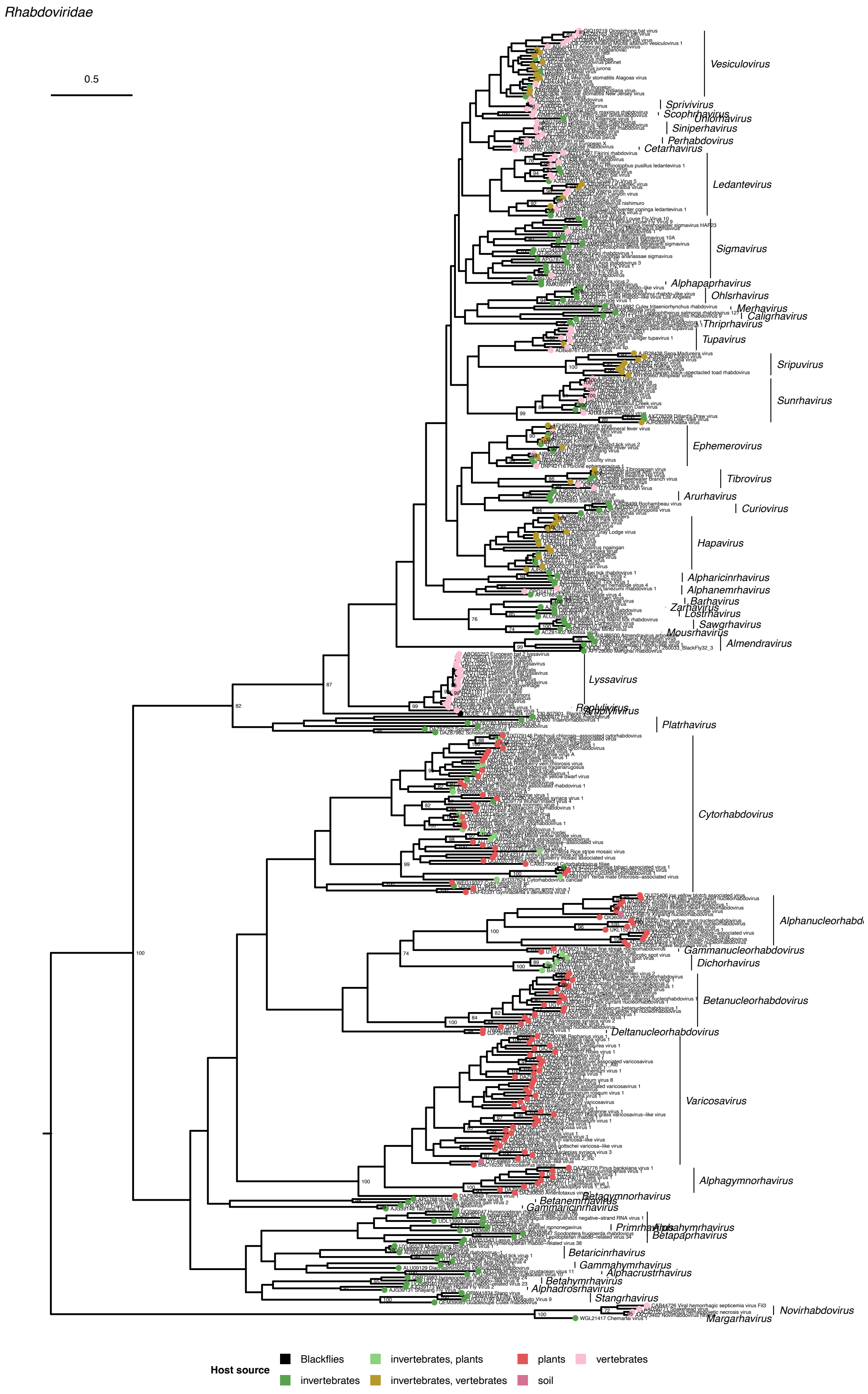

Supplementary Figure 14

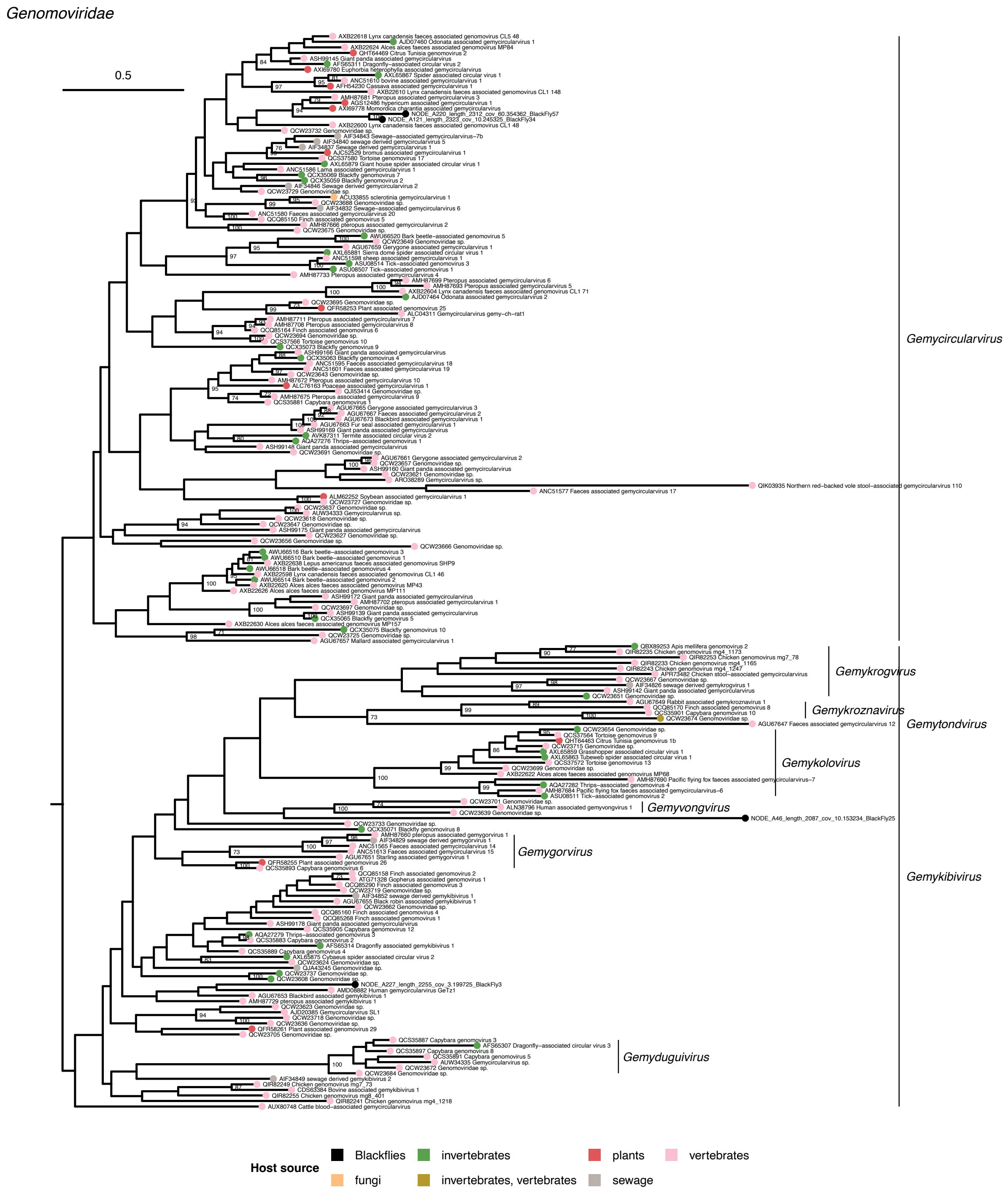

Supplementary Figure 15

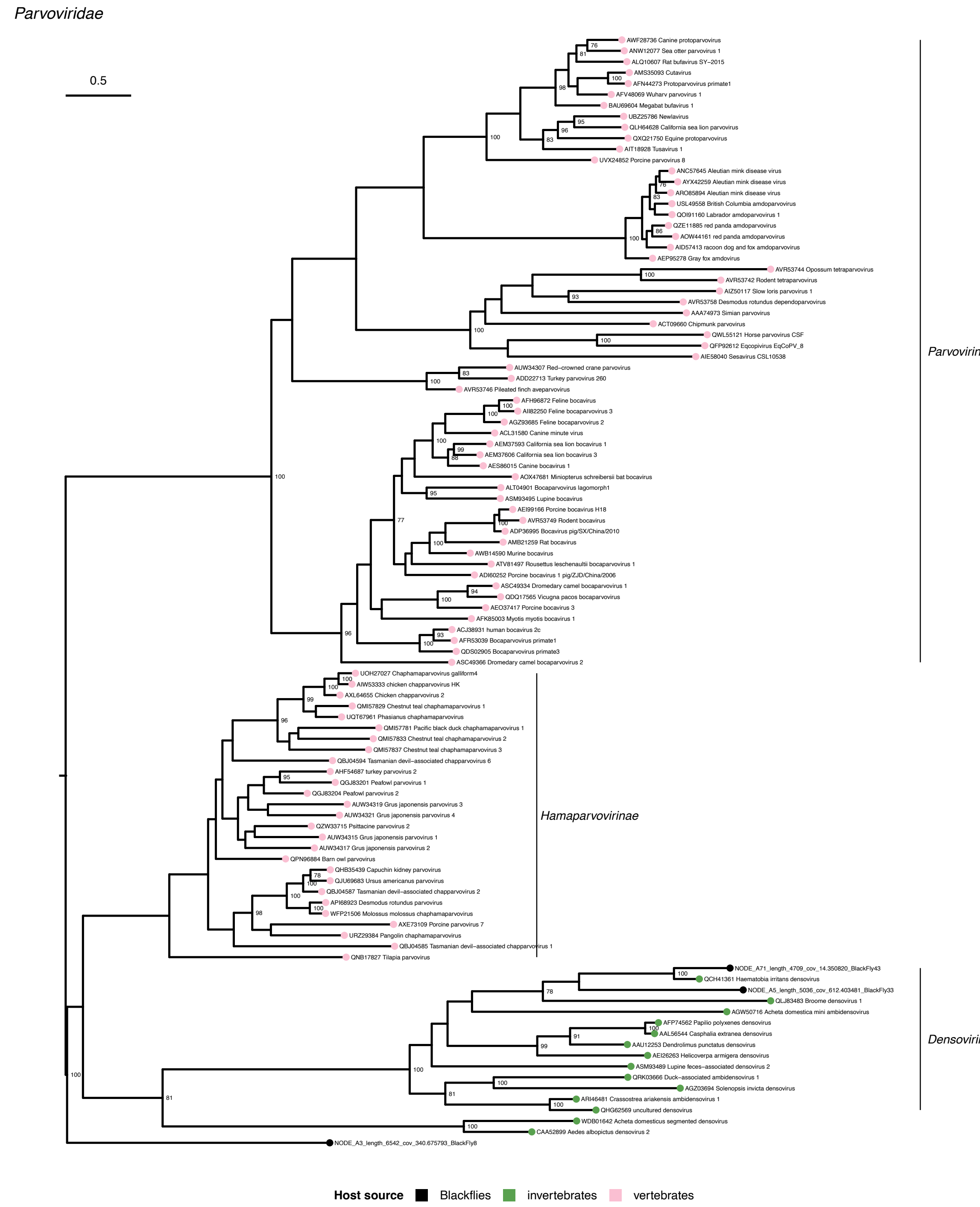

Supplementary Figure 16

Supplementary Table 1

Sample	num_seqs_raw	sum_len_raw	num_seqs_trimmed	sum_len_trimmed
**BlackFly3**	15,967,058	2,411,025,758	14,083,411	1,802,680,773
BlackFly4	10,618,738	1,603,429,438	9,517,543	1,192,269,936
BlackFly5	16,150,320	2,438,698,320	14,102,123	1,717,230,119
BlackFly6	12,647,152	1,909,719,952	11,036,288	1,353,645,999
BlackFly7	9,975,134	1,506,245,234	8,399,644	1,023,297,094
BlackFly8	9,969,112	1,505,335,912	7,795,933	931,437,183
BlackFly9	13,861,100	2,093,026,100	11,036,751	1,253,421,775
BlackFly10	9,500,948	1,434,643,148	7,377,393	851,224,264
BlackFly11	11,569,104	1,746,934,704	8,932,398	1,016,081,035
BlackFly12	17,760,656	2,681,859,056	16,170,603	2,017,817,725
BlackFly13	11,621,738	1,754,882,438	9,761,262	1,121,529,459
BlackFly14	13,825,740	2,087,686,740	11,367,218	1,368,627,775
BlackFly15	12,254,150	1,850,376,650	10,898,369	1,343,794,670
BlackFly16	23,262,772	3,512,678,572	20,790,370	2,593,968,097
BlackFly17	8,487,868	1,281,668,068	6,552,560	710,985,522
BlackFly18	11,315,546	1,708,647,446	8,810,100	975,232,370
BlackFly19	17,325,276	2,616,116,676	14,611,737	1,783,268,241
BlackFly20	21,282,790	3,213,701,290	18,469,265	2,287,470,708
BlackFly21	11,482,716	1,733,890,116	9,342,539	1,068,919,360
BlackFly22	7,261,050	1,096,418,550	5,272,569	563,885,155
BlackFly23	18,917,278	2,856,508,978	16,131,017	2,005,350,235
BlackFly24	9,540,220	1,440,573,220	7,108,174	752,892,879
BlackFly25	12,024,060	1,815,633,060	9,368,749	1,076,209,165
BlackFly26	9,754,940	1,472,995,940	7,576,311	859,096,268
BlackFly27	18,857,860	2,847,536,860	15,629,349	1,884,197,878
BlackFly28	16,220,006	2,449,220,906	14,396,532	1,753,659,158
BlackFly29	19,156,540	2,892,637,540	16,901,664	2,088,463,894
BlackFly30	20,808,714	3,142,115,814	18,463,756	2,341,331,981
BlackFly31	21,630,972	3,266,276,772	19,174,474	2,436,102,195
BlackFly32	14,763,992	2,229,362,792	12,656,550	1,548,953,159
BlackFly33	6,113,870	923,194,370	4,898,036	528,128,022
BlackFly34	24,002,232	3,624,337,032	20,578,254	2,601,183,937
BlackFly35	16,871,692	2,547,625,492	14,462,414	1,790,150,100
BlackFly36	14,992,036	2,263,797,436	12,568,209	1,551,430,241
BlackFly37	7,467,628	1,127,611,828	4,544,534	440,090,237
BlackFly38	10,610,832	1,602,235,632	7,890,352	900,521,109
BlackFly39	13,739,574	2,074,675,674	10,920,458	1,243,675,287
BlackFly40	8,646,086	1,305,558,986	6,611,682	683,202,413
BlackFly41	10,029,796	1,514,499,196	7,579,750	873,231,109
BlackFly42	14,691,830	2,218,466,330	12,196,809	1,443,227,901
BlackFly43	14,200,262	2,144,239,562	11,087,511	1,274,596,527
BlackFly44	12,190,272	1,840,731,072	9,998,501	1,147,085,995

BlackFly45	12,266,724	1,852,275,324	9,756,587	1,106,730,398
BlackFly46	10,546,382	1,592,503,682	7,436,299	778,570,975
BlackFly47	9,580,822	1,446,704,122	7,122,148	799,489,096
BlackFly48	11,021,364	1,664,225,964	9,059,033	1,015,534,398
BlackFly49	9,189,200	1,387,569,200	7,021,789	786,538,945
BlackFly50	13,864,316	2,093,511,716	12,268,048	1,420,847,975
BlackFly51	8,788,176	1,327,014,576	6,602,351	690,633,816
BlackFly52	12,479,582	1,884,416,882	10,616,888	1,243,645,802
BlackFly53	16,310,048	2,462,817,248	13,489,585	1,583,220,590
BlackFly55	8,522,448	1,286,889,648	6,572,453	715,669,985
BlackFly57	9,687,044	1,462,743,644	7,076,582	760,455,742
BlackFly58	9,418,760	1,422,232,760	6,903,583	751,600,122
BlackFly59	7,709,430	1,164,123,930	5,290,944	541,471,964

Supplementary Table 2

Accession	Family	Host	GenBank_Title
**NC_078466.1**	*Rhabdoviridae*	Trifolium pratense	Red clover varicosavirus isolate HZ2 segment RNA2, complete sequence
**NC_078467.1**	*Rhabdoviridae*	Trifolium pratense	Red clover varicosavirus isolate HZ2 segment RNA1, complete sequence
**NC_079051.1**	*Rhabdoviridae*		MAG: Xinjiang varicosa-like virus isolate 237-k141_63393 segment 1, complete sequence
**NC_079052.1**	*Rhabdoviridae*		MAG: Xinjiang varicosa-like virus isolate 237-k141_63393 segment 2, complete sequence
**NC_079119.1**	*Rhabdoviridae*	Brassica rapa	Brassica rapa virus 1 N (QK880_s2gp1), protein 2 (QK880_s2gp2), and protein 3 (QK880_s2gp3) genes, complete cds
**NC_079120.1**	*Rhabdoviridae*	Brassica rapa	Brassica rapa virus 1 L (QK880_s1gp1) gene, complete cds
**NC_079121.1**	*Rhabdoviridae*	Lolium perenne	Lolium perenne virus 1 N (QK881_s2gp1), protein 2 (QK881_s2gp2), and protein 3 (QK881_s2gp3) genes, complete cds
**NC_079122.1**	*Rhabdoviridae*	Lolium perenne	Lolium perenne virus 1 L (QK881_s1gp1) gene, complete cds
**NC_079123.1**	*Rhabdoviridae*	Melampyrum roseum	Melampyrum roseum virus 1 L (QK882_s1gp1) gene, complete cds
**NC_079124.1**	*Rhabdoviridae*	Melampyrum roseum	Melampyrum roseum virus 1 N (QK882_s2gp1), protein 2 (QK882_s2gp2), protein 3 (QK882_s2gp3), and protein 4 (QK882_s2gp4) genes, complete cds
**NC_079125.1**	*Rhabdoviridae*	Zostera marina	Zostera associated varicosavirus 1 isolate Zoma segment RNA2 nucleocapsid (N), 40K protein 2 (P2), 34K protein 3 (P3), and 23K protein 4 (P4) genes, complete cds
**NC_079126.1**	*Rhabdoviridae*	Zostera marina	Zostera associated varicosavirus 1 isolate Zoma segment RNA1 polymerase (L) gene, complete cds
**NC_079127.1**	*Rhabdoviridae*	Allium angulosum	Allium angulosum virus 1 protein L (QK884_s1gp1) gene, complete cds
**NC_079128.1**	*Rhabdoviridae*	Allium angulosum	Allium angulosum virus 1 protein N (QK884_s2gp1), protein 2 (QK884_s2gp2), and protein 3 (QK884_s2gp3) genes, complete cds
**NC_075304.1**	*Rhabdoviridae*	Culex	Culex rhabdo-like virus strain CRVL/Los Angeles, complete genome
**NC_075981.1**	*Rhabdoviridae*	Trichoprosopon theobaldi	Aruac virus nucleoprotein, phosphoprotein, matrix, glycoprotein, hypothetical proteins, and polymerase genes, complete cds
**NC_075982.1**	*Rhabdoviridae*	Ixodes dentatus	Connecticut virus nucleoprotein, phosphoprotein, hypothetical protein, matrix, glycoprotein, hypothetical protein, and polymerase genes, complete cds
**NC_075990.1**	*Rhabdoviridae*	Nyssomyia flaviscutellata	Inhangapi virus nucleoprotein, phosphoprotein, matrix, glycoprotein, hypothetical protein, and polymerase genes, complete cds
**NC_076030.1**	*Rhabdoviridae*	Phlebotomus	Charleville virus strain Ch9824, partial genome
**NC_076145.1**	*Rhabdoviridae*	Drosophila sturtevanti	Drosophila sturtevanti sigmavirus nucleocapsid protein (N), polymerase-associated protein (P), PP3 (X), matrix protein (M), glycoprotein (G), and RNA-dependent RNA polymerase (L) genes, complete cds

**NC_076146.1**	*Rhabdoviridae*	Ceratitis capitata	Ceratitis capitata sigmavirus nucleocapsid protein (N), polymerase-associated protein (P), PP3 (X), matrix protein (M), glycoprotein (G), and RNA-dependent RNA polymerase (L) genes, complete cds
**NC_076157.1**	*Rhabdoviridae*	Nematoda	Shayang ascaridia galli virus 2 strain HC21241 hypothetical protein 1 (QKL27_gp1), hypothetical protein 2 (QKL27_gp2), hypothetical protein 3 (QKL27_gp3), hypothetical protein 4 (QKL27_gp4), putative glycoprotein (QKL27_gp5), and RNA-dependent RNA polymerase (QKL27_gp6) genes, complete cds
**NC_076163.1**	*Rhabdoviridae*	Solanum tuberosum	Potato yellow dwarf nucleorhabdovirus strain CYDV-constricta, complete genome
**NC_076182.1**	*Rhabdoviridae*	Culex pseudovishnui	Culex pseudovishnui rhabdo-like virus 17NGK-Cps2-874 genes for nucleoprotein, phosphoprotein, hyppothetical protein, glycoprotein, RNA-dependent RNA polymerase, complete cds
**NC_076208.1**	*Rhabdoviridae*	Ixodes scapularis	New Kent County virus isolate RTS126, complete genome
**NC_076211.1**	*Rhabdoviridae*	Culex tarsalis	Dillard's Draw virus isolate DDrV-2015, complete genome
**NC_076239.1**	*Rhabdoviridae*	Fragaria	Cytorhabdovirus fragariarugosus isolate A, complete genome
**NC_076244.1**	*Rhabdoviridae*	Zanthoxylum	Green Sichuan pepper nucleorhabdovirus isolate ZPNu1, complete genome
**NC_076250.1**	*Polycipiviridae*	Lasius neglectus	Lasius neglectus virus 2, complete genome
**NC_076260.1**	*Rhabdoviridae*	Mansonia uniformis	Puchong virus isolate P5-350 Malaysian, complete genome
**NC_076261.1**	*Rhabdoviridae*	Bos indicus	Hayes Yard virus isolate DPP4816, complete genome
**NC_076267.1**	*Rhabdoviridae*	Malus domestica	Apple rootstock virus A, complete genome
**NC_076289.1**	*Rhabdoviridae*	Trifolium pratense	Trifolium pratense virus B isolate 1/2014 putative N protein (QKM62_gp1), putative P protein (QKM62_gp2), putative P3 protein (QKM62_gp3), putative M protein (QKM62_gp4), putative G protein (QKM62_gp5), and putative L protein (QKM62_gp6) genes, complete cds
**NC_076290.1**	*Rhabdoviridae*	Trifolium pratense	Trifolium pratense virus A isolate 29/15/1 putative N protein (QKM63_gp1), putative P protein (QKM63_gp2), putative P3 protein (QKM63_gp3), putative M protein (QKM63_gp4), putative G protein (QKM63_gp5), and putative L protein (QKM63_gp6) genes, complete cds
**NC_076400.1**	*Rhabdoviridae*	Psorophora albigenu	UNVERIFIED: Lobeira virus isolate BR/MT_M05 polyprotein-like gene, partial sequence
**NC_076415.1**	*Rhabdoviridae*	Amblyomma ovale	Blanchseco virus isolate TTP-Pool-17, complete genome
**NC_076430.1**	*Rhabdoviridae*	Elettaria cardamomum	Cardamom vein clearing nucleorhabdovirus 1, complete genome
**NC_076448.1**	*Rhabdoviridae*	Prunus persica	Peach virus 1 isolate NSTT, complete genome
**NC_076472.1**	*Rhabdoviridae*	Ilex paraguariensis	Yerba mate virus A isolate Gob. Virasoro, complete genome
**NC_076487.1**	*Rhabdoviridae*	Chlorion hirtum	Hymenopteran rhabdo-related virus OKIAV109 genomic sequence
**NC_076489.1**	*Rhabdoviridae*	Pompilus cinereus	Hymenopteran rhabdo-related virus OKIAV38 genomic sequence
**NC_076490.1**	*Rhabdoviridae*	Triodia sylvina	Lepidopteran rhabdo-related virus OKIAV34 genomic sequence
**NC_076494.1**	*Rhabdoviridae*	Dipseliopoda	Bughendera virus isolate BF402 nucleoprotein (QKO67_gp1), phosphoprotein (QKO67_gp2), matrix protein (QKO67_gp3), glycoprotein (QKO67_gp4), and large protein (QKO67_gp5) genes, complete cds
**NC_076501.1**	*Rhabdoviridae*	Heterodontonyx	Hymenopteran rhabdo-related virus OKIAV24 nucleoprotein (QKO74_gp1), hypothetical protein (QKO74_gp2), hypothetical protein (QKO74_gp3), glycoprotein (QKO74_gp4), and RdRp (QKO74_gp5) genes, complete cds
**NC_076510.1**	*Rhabdoviridae*	Sonchus oleraceus	Sowthistle yellow vein virus isolate HWY65, complete genome
**NC_076531.1**	*Rhabdoviridae*	Bacopa monnieri	Bacopa monnieri virus 1 isolate India, complete genome
**NC_076532.1**	*Rhabdoviridae*	Bacopa monnieri	Bacopa monnieri virus 2 isolate India, complete genome
**NC_076534.1**	*Rhabdoviridae*		Anole lyssa-like virus 1 A.allogus/Cuba/2011 RNA, complete genome
**NC_076686.1**	*Rhabdoviridae*	Dryophytes cinereus	Frog lyssa-like virus 1 strain FLLV1-MaleA, complete genome
**NC_076841.1**	*Rhabdoviridae*	Rhinolophus sinicus	Taiyi bat virus isolate 958, complete genome
**NC_076842.1**	*Rhabdoviridae*	Rhinolophus sinicus	Yinshui bat virus isolate 1017, complete genome
**NC_076864.1**	*Rhabdoviridae*		Paper mulberry mosaic-associated virus isolate SWU, complete genome

**NC_076909.1**	*Rhabdoviridae*	Rosa hybrid cultivar	Rose virus R isolate MDR92016, complete genome
**NC_076913.1**	*Rhabdoviridae*	Solanum aculeatissimum	Joa yellow blotch virus isolate Manaus, complete genome
**NC_076914.1**	*Rhabdoviridae*	Chrysanthemum x morifolium	Chrysanthemum yellow dwarf virus isolate cq, complete genome
**NC_076926.1**	*Rhabdoviridae*	Chrysura austriaca	Hymenopteran rhabdo-related virus isolate OKIAV23, complete sequence
**NC_076927.1**	*Rhabdoviridae*	Chrysura radians	Hymenopteran rhabdo-related virus isolate OKIAV46, complete sequence
**NC_076929.1**	*Rhabdoviridae*	Mansonia uniformis	Porton's virus isolate 0416MAL, partial genome
**NC_076930.1**	*Rhabdoviridae*	Culex perfuscus	Bangoran virus isolate 0424RCA, partial genome
**NC_076931.1**	*Rhabdoviridae*	Coquillettidia maculipennis	Boteke virus isolate 0417RCA, complete genome
**NC_076932.1**	*Rhabdoviridae*		Sandjimba virus isolate 0408RCA, complete genome
**NC_076933.1**	*Rhabdoviridae*	Eurillas virens	Nasoule virus isolate 0410RCA, complete genome
**NC_076934.1**	*Rhabdoviridae*		Bimbo virus isolate 9716RCA, complete genome
**NC_076935.1**	*Rhabdoviridae*		Kolongo virus isolate 9717RCA, complete genome
**NC_076936.1**	*Rhabdoviridae*	Ploceus melanocephalus	Ouango virus isolate 9718RCA, complete genome
**NC_076937.1**	*Rhabdoviridae*	Curruca curruca	Burg el Arab virus isolate 09023EGY, complete genome
**NC_076938.1**	*Rhabdoviridae*	Curruca curruca	Matariya virus isolate 09027EGY, complete genome
**NC_076939.1**	*Rhabdoviridae*	Rhinolophus ferrumequinum	Mediterranean bat virus isolate A09181, complete genome
**NC_076970.1**	*Rhabdoviridae*	Agave tequilana	Agave tequilana virus 1 N (QKT51_gp1), P (QKT51_gp2), P3 (QKT51_gp3), M (QKT51_gp4), G (QKT51_gp5), and L (QKT51_gp6) genes, complete cds
**NC_076971.1**	*Rhabdoviridae*	Asclepias syriaca	Asclepias syriaca virus 1 N (QKT52_gp1), P (QKT52_gp2), P3 (QKT52_gp3), M (QKT52_gp4), G (QKT52_gp5), P6 (QKT52_gp6), and L (QKT52_gp7) genes, complete cds
**NC_076972.1**	*Rhabdoviridae*	Asclepias syriaca	Asclepias syriaca virus 2 N (QKT53_gp1), P (QKT53_gp2), P3 (QKT53_gp3), M (QKT53_gp4), G (QKT53_gp5), and L (QKT53_gp6) genes, complete cds
**NC_076973.1**	*Rhabdoviridae*	Plectranthus aromaticus	Plectranthus aromaticus virus 1 N (QKT54_gp1), P (QKT54_gp2), P3 (QKT54_gp3), M (QKT54_gp4), G (QKT54_gp5), and L (QKT54_gp6) genes, complete cds
**NC_076974.1**	*Rhabdoviridae*	Rhododendron delavayi	Rhododendron delavayi virus 1 N (QKT55_gp1), P (QKT55_gp2), P3 (QKT55_gp3), M (QKT55_gp4), G (QKT55_gp5), and L (QKT55_gp6) genes, complete cds
**NC_076975.1**	*Rhabdoviridae*	Anthurium amnicola	Anthurium amnicola virus 1 N (QKT56_gp1), P (QKT56_gp2), P3 (QKT56_gp3), M (QKT56_gp4), G (QKT56_gp5), and L (QKT56_gp6) genes, complete cds
**NC_076976.1**	*Rhabdoviridae*	Bemisia tabaci	Bemisia tabaci associated virus 1 N (QKT57_gp1), P (QKT57_gp2), P3 (QKT57_gp3), M (QKT57_gp4), G (QKT57_gp5), and L (QKT57_gp6) genes, complete cds
**NC_076977.1**	*Rhabdoviridae*	Glehnia littoralis	Glehnia littoralis virus 1 N (QKT58_gp1), P (QKT58_gp2), P3 (QKT58_gp3), M (QKT58_gp4), G (QKT58_gp5), P6 (QKT58_gp6), and L (QKT58_gp7) genes, complete cds
**NC_076978.1**	*Rhabdoviridae*	Gymnadenia x densiflora	Gymnadenia x densiflora virus 1 N (QKT59_gp1), P (QKT59_gp2), M (QKT59_gp3), and L (QKT59_gp4) genes, complete cds
**NC_076979.1**	*Rhabdoviridae*	Nymphaea alba	Nymphaea alba virus 1 N (QKT60_gp1), P (QKT60_gp2), P3 (QKT60_gp3), M (QKT60_gp4), G (QKT60_gp5), P6 (QKT60_gp6), and L (QKT60_gp7) genes, complete cds
**NC_076980.1**	*Rhabdoviridae*	Tagetes erecta	Tagetes erecta virus 1 N (QKT61_gp1), P (QKT61_gp2), P3 (QKT61_gp3), M (QKT61_gp4), and L (QKT61_gp5) genes, complete cds
**NC_076981.1**	*Rhabdoviridae*	Trachyspermum ammi	Trachyspermum ammi virus 1 N (QKT62_gp1), P (QKT62_gp2), P3 (QKT62_gp3), M (QKT62_gp4), and L (QKT62_gp5) genes, complete cds
**NC_076982.1**	*Rhabdoviridae*	Pinus flexilis	Pinus flexilis virus 1 N (QKT63_gp1), protein 2 (QKT63_gp2), protein 3 (QKT63_gp3), protein 4 (QKT63_gp4), and L (QKT63_gp5) genes, complete cds
**NC_077111.1**	*Rhabdoviridae*	Trichobius sp.	Mejal virus isolate JAL10 nucleoprotein (N), phosphoprotein (P), matrix protein (M), glycoprotein (G), and RNA-dependent RNA polymerase (L) genes, complete cds
**NC_077114.1**	*Rhabdoviridae*	Aves	Rhabdoviridae sp. isolate YSN900 genomic sequence
**NC_077123.1**	*Rhabdoviridae*	Frankliniella intonsa	MAG: Hangzhou frankliniella intonsa rhabdovirus 1 isolate JM1FY86115, complete genome
**NC_077129.1**	*Rhabdoviridae*	Rhinolophus pearsonii	Wufeng Rhinolophus pearsonii tupavirus 1, complete genome

**NC_077130.1**	*Rhabdoviridae*	Leopoldamys edwardsi	Longquan Niviventer coninga ledantevirus 1, complete genome
**NC_077151.1**	*Rhabdoviridae*	Cnidium officinale	Cnidium virus 1 isolate SK, complete genome
**NC_077158.1**	*Rhabdoviridae*	Insecta	MAG: Xiangshan rhabdo-like virus 1 isolate Novel_23 nucleocapsid protein, hypothetical protein, spike glycoprotein, and RNA dependent RNA polymerase genes, complete cds
**NC_077192.1**	*Rhabdoviridae*	Homo sapiens	Mundri virus isolate A14, partial genome
**NC_055137.1**	*Rhabdoviridae*	Lepeophtheirus salmonis	Lepeophtheirus salmonis rhabdovirus No9, partial genome
**NC_055138.1**	*Rhabdoviridae*	Lepeophtheirus salmonis	Lepeophtheirus salmonis rhabdovirus No127, partial genome
**NC_055208.1**	*Rhabdoviridae*	Citrus sinensis	Citrus chlorotic spot virus strain Trs1 segment RNA1, complete sequence
**NC_055290.1**	*Rhabdoviridae*	Culex sitiens	North Creek virus phosphoprotein (P) gene, partial cds
**NC_055291.1**	*Rhabdoviridae*	Culex sitiens	North Creek virus glycoprotein (G) gene, complete cds
**NC_055292.1**	*Rhabdoviridae*	Culex sitiens	North Creek virus RNA dependent RNA polymerase (L) gene, complete cds
**NC_055293.1**	*Rhabdoviridae*	Culex sitiens	North Creek virus nucleoprotein (N) gene, partial cds
**NC_055454.1**	*Rhabdoviridae*	Zea mays	Maize yellow striate virus, complete genome
**NC_055456.1**	*Rhabdoviridae*	Culex	Kwatta virus nucleoprotein, phosphoprotein, hypothetical protein, matrix, hypothetical protein, glycoprotein, and polymerase genes, complete cds
**NC_055457.1**	*Rhabdoviridae*	Haemaphysalis leporispalustris	New Minto virus nucleoprotein, phosphoprotein, matrix, glycoprotein, and polymerase genes, complete cds
**NC_055460.1**	*Rhabdoviridae*	Culex annulirostris	Harrison Dam virus isolate CS75, partial genome
**NC_055461.1**	*Rhabdoviridae*	Dermacentor variabilis	Sawgrass virus nucleoprotein, phosphoprotein, matrix, glycoprotein, and polymerase genes, complete cds
**NC_055466.1**	*Rhabdoviridae*	Physostegia	Physostegia chlorotic mottle virus isolate PV-1182, complete genome
**NC_055473.1**	*Rhabdoviridae*	Culex annulirostris	Holmes Jungle virus isolate DPP1163, complete genome
**NC_055474.1**	*Rhabdoviridae*	Pipistrellus abramus	Taiwan bat lyssavirus isolate TWBLV/TN/2016, complete genome
**NC_055477.1**	*Rhabdoviridae*	Ochlerotatus cantans	Ohlsdorf virus strain Germany/2012/Oc.cantans, complete genome
**NC_055479.1**	*Rhabdoviridae*	Brassica oleracea	Cabbage cytorhabdovirus 1 strain FERA_050726, complete genome
**NC_055484.1**	*Rhabdoviridae*	Triticum aestivum	Wheat yellow striate virus isolate SX-HC nucleocapsid protein, putative phosphoprotein, P3 protein, matrix protein, glycoprotein, hypothetical protein P6, and probable RNA-dependent RNA polymerase genes, complete cds
**NC_055504.1**	*Rhabdoviridae*	Carica papaya	Papaya cytorhabdovirus isolate Los Rios_Ec, complete genome
**NC_055505.1**	*Rhabdoviridae*	Ilex paraguariensis	Yerba mate chlorosis-associated virus isolate Montecarlo, complete genome
**NC_055509.1**	*Rhabdoviridae*	Rhinella marina	Cuiaba virus strain BeAn 227841, partial genome
**NC_055512.1**	*Rhabdoviridae*	Zea mays	Morogoro maize-associated virus isolate 16-0112 nucleocapsid protein, phosphoprotein, putative movement protein, matrix protein, glycoprotein, and RNA-dependent RNA polymerase genes, complete cds
**NC_055529.1**	*Rhabdoviridae*	Rubus idaeus	Raspberry vein chlorosis virus isolate Hutton_1, complete genome
**NC_055530.1**	*Rhabdoviridae*	Corythornis cristatus	Garba virus nucleoprotein, phosphoprotein, hypothetical protein, matrix, hypothetical protein, glycoprotein, and polymerase genes, complete cds
**NC_055531.1**	*Rhabdoviridae*	Ochlerotatus sollicitans	Bahia Grande virus nucleoprotein, phosphoprotein, matrix, glycoprotein, hypothetical protein, and polymerase genes, complete cds
**NC_055532.1**	*Rhabdoviridae*	Aedes sp.	Muir Springs virus nucleoprotein, phosphoprotein, matrix, glycoprotein, and polymerase genes, complete cds
**NC_055567.1**	*Rhabdoviridae*	Fragaria x ananassa	Strawberry cytorhabdovirus 1 isolate B, complete genome
**NC_052231.1**	*Rhabdoviridae*	Citrus sinensis	Citrus leprosis virus N strain ibi1 segment RNA2, complete sequence
**NC_043065.1**	*Rhabdoviridae*	Drosophila tristis	Drosophila tristis sigmavirus RNA-dependent RNA polymerase (L) gene, partial cds
**NC_043066.1**	*Rhabdoviridae*	Muscina stabulans	Muscina stabulans sigma virus RNA-dependent RNA polymerase (L) gene, partial cds
**NC_043067.1**	*Rhabdoviridae*	Homo sapiens	Bas-Congo virus isolate BASV-1 N protein gene, partial cds

**NC_043525.1**	*Rhabdoviridae*	Caligus rogercresseyi	Caligus rogercresseyi rhabdovirus strain CrRV-Ch01, partial genome
**NC_043538.1**	*Rhabdoviridae*	Pipistrellus kuhlii	Vaprio virus nucleoprotein, phosphoprotein, matrix, glycoprotein, transcriptional unit 1, and polymerase genes, complete cds
**NC_043649.1**	*Rhabdoviridae*	Clerodendrum sp.	Clerodendrum chlorotic spot virus isolate Prb1 segment RNA2, complete sequence
**NC_040599.1**	*Rhabdoviridae*	Culex quinquefasciatus	Merida virus isolate MERD-Mex07, complete genome
**NC_040602.1**	*Rhabdoviridae*	Aedes albopictus	Menghai rhabdovirus isolate Menghai, complete genome
**NC_040664.1**	*Rhabdoviridae*	Hyalomma anatolicum anatolicum	Zahedan rhabdovirus isolate Ar Teh 157764, complete genome
**NC_040669.1**	*Rhabdoviridae*	Ochlerotatus sp.	Riverside virus 1 strain RISV-Drava 1, complete genome
**NC_040786.1**	*Rhabdoviridae*	Oryza sativa	Rice stripe mosaic virus isolate GD-LD, complete genome
**NC_038236.1**	*Rhabdoviridae*	Equus caballus	Vesicular stomatitis Indiana virus strain 98COE, complete genome
**NC_038275.1**	*Rhabdoviridae*	Culex sitiens	Mossuril virus nucleoprotein, phosphoprotein, hypothetical proteins, matrix, glycoprotein, hypothetical protein, and polymerase genes, complete cds
**NC_038276.1**	*Rhabdoviridae*	Sus scrofa	Nishimuro virus viral cRNA for hypothetical proteins, complete cds
**NC_038277.1**	*Rhabdoviridae*	Salmo trutta	Trout rhabdovirus 903/87 nucleocapsid protein, phosphoprotein, matrix protein, and glycoprotein genes, complete cds
**NC_038278.1**	*Rhabdoviridae*	Drosophila affinis	Drosophila affinis sigmavirus nucleocapsid protein (N), polymerase-associated protein (P), PP3 (X), matrix protein (M), glycoprotein (G), and RNA-dependent RNA polymerase (L) genes, complete cds
**NC_038279.1**	*Rhabdoviridae*	Drosophila ananassae	Drosophila ananassae sigmavirus nucleocapsid protein (N), polymerase-associated protein (P), PP3 (X), matrix protein (M), glycoprotein (G), and RNA-dependent RNA polymerase (L) genes, complete cds
**NC_038280.1**	*Rhabdoviridae*	Diptera	Drosophila immigrans sigmavirus strain SCM45623 nucleocapsid protein, polymerase-associated protein, PP3, matrix protein, glycoprotein, and RNA-dependent RNA polymerase genes, complete cds
**NC_038281.1**	*Rhabdoviridae*	Drosophila melanogaster	Drosophila melanogaster sigma virus HAP23, complete genome
**NC_038282.1**	*Rhabdoviridae*	Homo sapiens	Ekpoma virus 1 isolate EKV-1, partial genome
**NC_038283.1**	*Rhabdoviridae*	Homo sapiens	Ekpoma virus 2 isolate EKV-2, partial genome
**NC_038284.1**	*Rhabdoviridae*	Aves	Durham virus nucleocapsid, phosphoprotein, putative protein C, matrix protein, small hydrophobic protein, and glycoprotein genes, complete cds
**NC_038285.1**	*Rhabdoviridae*	Lutzomyia	Carajas virus nucleoprotein, phosphoprotein, matrix, glycoprotein, and polymerase genes, complete cds
**NC_038286.1**	*Rhabdoviridae*	Philander opossum	Piry virus strain BeAn2423, complete genome
**NC_038287.1**	*Rhabdoviridae*	Phlebotomus perfiliewi	Radi virus nucleoprotein, phosphoprotein, matrix, glycoprotein, and polymerase genes, complete cds
**NC_038755.1**	*Rhabdoviridae*		Coffee ringspot virus strain Lavras segment RNA2, complete sequence
**NC_039020.1**	*Rhabdoviridae*	Dipseliopoda	Kanyawara virus isolate MPK004 nucleoprotein, phosphoprotein, matrix, glycoprotein, and polymerase genes, complete cds
**NC_039021.1**	*Rhabdoviridae*	Culicoides peregrinus	Beatrice Hill virus isolate CSIRO 25, complete genome
**NC_039200.1**	*Rhabdoviridae*	Psorophora albigenu	Balsa almendravirus, complete genome
**NC_039201.1**	*Rhabdoviridae*	Culicoides	Curionopolis virus nucleoprotein, phosphoprotein, matrix, hypothetical proteins, glycoprotein, hypothetical proteins, and polymerase genes, complete cds
**NC_039202.1**	*Rhabdoviridae*	Culiseta melanura	Flanders virus nucleoprotein, phosphoprotein, hypothetical proteins, matrix, glycoprotein, hypothetical protein, and polymerase genes, complete cds
**NC_039206.1**	*Rhabdoviridae*	Haemagogus	Jurona virus nucleoprotein, phosphoprotein, matrix, glycoprotein, and polymerase genes, complete cds
**NC_036390.1**	*Rhabdoviridae*	Zea mays	Maize Iranian mosaic nucleorhabdovirus, complete genome
**NC_031957.1**	*Rhabdoviridae*	Anopheles quadrimaculatus	Coot Bay virus strain EVG5-53, complete sequence
**NC_031958.1**	*Rhabdoviridae*	Culicidae	Rio Chico virus strain GAM 195, complete sequence

**NC_035132.1**	*Rhabdoviridae*	Culex quinquefasciatus	Culex rhabdo-like virus strain mosWSB71420, complete genome
**NC_034508.1**	*Rhabdoviridae*	Lutzomyia	Morreton virus nucleoprotein, phosphoprotein, matrix, glycoprotein, and polymerase genes, complete cds
**NC_034529.1**	*Rhabdoviridae*	Ameiva ameiva	Sena Madureira virus nucleoprotein, hypothetical protein, phosphoprotein, matrix, glycoprotein, hypothetical protein, and polymerase genes, complete cds
**NC_034530.1**	*Rhabdoviridae*	Ameiva ameiva	Marco virus nucleoprotein, phosphoprotein, matrix, glycoprotein, hypothetical proteins, and polymerase genes, complete cds
**NC_034531.1**	*Rhabdoviridae*	Culex tarsalis	Manitoba virus nucleoprotein, phosphoprotein, hypothetical proteins, matrix, glycoprotein, hypothetical protein, and polymerase genes, complete cds
**NC_034533.1**	*Rhabdoviridae*	Riparia paludicola	Landjia virus nucleoprotein, phosphoprotein, hypothetical proteins, matrix, glycoprotein, hypothetical proteins, and polymerase genes, complete cds
**NC_034534.1**	*Rhabdoviridae*	Coquillettidia albicosta	Rochambeau virus nucleoprotein, phosphoprotein, matrix, hypothetical proteins, glycoprotein, hypothetical proteins, and polymerase genes, complete cds
**NC_034535.1**	*Rhabdoviridae*	Rattus rattus	Barur virus nucleoprotein, phosphoprotein, matrix, glycoprotein, and polymerase genes, complete cds
**NC_034536.1**	*Rhabdoviridae*	Culicoides	Itacaiunas virus nucleoprotein, phosphoprotein, matrix, hypothetical protein, glycoprotein, hypothetical protein, and polymerase genes, complete cds
**NC_034537.1**	*Rhabdoviridae*	Culex dunni	La Joya virus nucleoprotein, phosphoprotein, hypothetical proteins, matrix, hypothetical proteins, glycoprotein, hypothetical proteins, and polymerase genes, complete cds
**NC_034538.1**	*Rhabdoviridae*	Culicinae	Joinjakaka virus nucleoprotein, phosphoprotein, hypothetical protein, matrix, glycoprotein, hypothetical proteins, and polymerase genes, complete cds
**NC_034539.1**	*Rhabdoviridae*	Eretmapodites leucopous	Nkolbisson virus nucleoprotein, phosphoprotein, matrix, glycoprotein, and polymerase genes, complete cds
**NC_034540.1**	*Rhabdoviridae*	Gerbilliscus kempi	Keuraliba virus nucleoprotein, phosphoprotein, matrix, glycoprotein, hypothetical protein, and polymerase genes, complete cds
**NC_034541.1**	*Rhabdoviridae*	Culex tarsalis	Gray Lodge virus nucleoprotein, phosphoprotein, hypothetical proteins, matrix, glycoprotein, hypothetical protein, and polymerase genes, complete cds
**NC_034542.1**	*Rhabdoviridae*	Sergentomyia	Sripur virus nucleoprotein, hypothetical protein, phosphoprotein, hypothetical protein, matrix, hypothetical protein, glycoprotein, hypothetical protein, and polymerase genes, complete cds
**NC_034543.1**	*Rhabdoviridae*	Culex annulirostris	Ord River virus nucleoprotein, hypothetical protein, phosphoprotein, hypothetical proteins, matrix, glycoprotein, hypothetical protein, and polymerase genes, complete cds
**NC_034544.1**	*Rhabdoviridae*	Lutzomyia	Iriri virus nucleoprotein, phosphoprotein, matrix, hypothetical proteins, glycoprotein, hypothetical proteins, and polymerase genes, complete cds
**NC_034545.1**	*Rhabdoviridae*	Rhinolophus eloquens	Mount Elgon bat virus nucleoprotein, phosphoprotein, matrix, glycoprotein, and polymerase genes, complete cds
**NC_034546.1**	*Rhabdoviridae*	Culicoides insignis	Sweetwater Branch virus nucleoprotein, phosphoprotein, matrix, hypothetical proteins, glycoprotein, hypothetical protein, and polymerase genes, complete cds
**NC_034548.1**	*Rhabdoviridae*	Rhinolophus cornutus	Oita virus nucleoprotein, phosphoprotein, matrix, glycoprotein, and polymerase genes, complete cds
**NC_034549.1**	*Rhabdoviridae*	Microtus montanus	Klamath virus nucleoprotein, phosphoprotein, hypothetical protein, matrix, hypothetical protein, glycoprotein, hypothetical protein, and polymerase genes, complete cds
**NC_034550.1**	*Rhabdoviridae*	Ameiva ameiva	Chaco virus nucleoprotein, hypothetical protein, phosphoprotein, matrix, hypothetical protein, glycoprotein, hypothetical protein, and polymerase genes, complete cds
**NC_034551.1**	*Rhabdoviridae*	Colocasia esculenta	Colocasia bobone disease-associated virus strain SI, complete genome
**NC_034443.1**	*Rhabdoviridae*	Homo sapiens	Le Dantec virus nucleoprotein, phosphoprotein, matrix, glycoprotein, hypothetical protein, and polymerase genes, complete cds
**NC_034447.1**	*Rhabdoviridae*	Culex tarsalis	Hart Park virus nucleoprotein, phosphoprotein, hypothetical proteins, matrix, glycoprotein, hypothetical protein, and polymerase genes, complete cds
**NC_034448.1**	*Rhabdoviridae*	Culex portesi	Mosqueiro virus nucleoprotein, phosphoprotein, hypothetical proteins, matrix, glycoprotein, hypothetical protein, and polymerase genes, complete cds
**NC_034449.1**	*Rhabdoviridae*	Culex annulirostris	Parry Creek virus nucleoprotein, phosphoprotein, hypothetical proteins, matrix, glycoprotein, hypothetical protein, and polymerase genes, complete cds
**NC_034450.1**	*Rhabdoviridae*	Culex annulioris	Kamese virus nucleoprotein, phosphoprotein, hypothetical proteins, matrix, glycoprotein, hypothetical protein, and polymerase genes, complete cds
**NC_034451.1**	*Rhabdoviridae*	Myotis yumanensis	Kern Canyon virus nucleoprotein, phosphoprotein, matrix, glycoprotein, hypothetical protein, and polymerase genes, complete cds
**NC_034454.1**	*Rhabdoviridae*	Culicoides punctatus	Fukuoka virus nucleoprotein, phosphoprotein, matrix, hypothetical protein, glycoprotein, and polymerase genes, complete cds
**NC_034240.1**	*Rhabdoviridae*	Solanum lycopersicum	Tomato yellow mottle-associated virus, complete genome

**NC_033701.1**	*Rhabdoviridae*	Nematoda	Xingshan nematode virus 4 strain XSNXC32924 putative nucleoprotein, hypothetical protein 1, hypothetical protein 2, putative glycoprotein, and RNA-dependent RNA polymerase genes, complete cds
**NC_033705.1**	*Rhabdoviridae*	Nematoda	Xinzhou nematode virus 4 strain XZSJSC65771 putative nucleoprotein, hypothetical protein 2, hypothetical protein 3, putative glycoprotein, and RNA-dependent RNA polymerase genes, complete cds
**NC_032739.1**	*Rhabdoviridae*	Crustacea	Wenling crustacean virus 10 strain WLJQ101844 hypothetical protein 1, hypothetical protein 2, hypothetical protein 3, putative glycoprotein, and RNA-dependent RNA polymerase genes, complete cds
**NC_032781.1**	*Rhabdoviridae*	Crustacea	Wenling crustacean virus 11 strain WLJQ201798 hypothetical protein 1, hypothetical protein 2, hypothetical protein 3, putative glycoprotein, and RNA-dependent RNA polymerase genes, complete cds
**NC_032907.1**	*Rhabdoviridae*	Lepidoptera	Hubei lepidoptera virus 2 strain LCM101902 putative nucleoprotein, hypothetical protein 2, putative membrane protein, putative glycoprotein 1, putative glycoprotein 2, and RNA-dependent RNA polymerase genes, complete cds
**NC_033034.1**	*Rhabdoviridae*	Diptera	Hubei diptera virus 9 strain SCM172232 putative nucleoprotein, hypothetical protein 2, putative X protein, putative matrix protein, putative glycoprotein, and RNA-dependent RNA polymerase genes, complete cds
**NC_033070.1**	*Rhabdoviridae*	Diptera	Hubei dimarhabdovirus virus 1 strain SCM51525 putative nucleoprotein, hypothetical protein, putative matrix protein, putative glycoprotein, and RNA-dependent RNA polymerase genes, complete cds
**NC_033103.1**	*Rhabdoviridae*	Diptera	Hubei diptera virus 10 strain SCM43656 putative nucleoprotein, hypothetical protein, putative X protein, putative matrix protein, putative glycoprotein, and RNA-dependent RNA polymerase genes, complete cds
**NC_033267.1**	*Rhabdoviridae*	Ascaris suum	Hubei rhabdo-like virus 9 strain WHZHC73015 hypothetical protein 1, hypothetical protein 2, hypothetical protein 3, hypothetical protein 4, hypothetical protein 5, putative glycoprotein, and RNA-dependent RNA polymerase genes, complete cds
**NC_031988.1**	*Rhabdoviridae*	Pteropus giganteus	Gannoruwa bat lyssavirus isolate RV3266, complete genome
**NC_031955.1**	*Rhabdoviridae*	Miniopterus schreibersii	Lleida bat lyssavirus isolate RV3208, complete genome
**NC_031301.1**	*Rhabdoviridae*	Hippoboscidae	Wuhan Louse Fly Virus 5 strain BFJSC-5 nucleocapsid (N), phosphoprotein (P), matrix protein (M), glycoprotein (G), and RNA-dependent RNA polymerase (L) genes, complete cds
**NC_031302.1**	*Rhabdoviridae*	Hippoboscidae	Wuhan Louse Fly Virus 9 strain BFJSC-7 nucleocapsid (N), putative phosphoprotein (ORF2), matrix protein (M), glycoprotein (G), and RNA-dependent RNA polymerase (L) genes, complete cds
**NC_031304.1**	*Rhabdoviridae*	Rhipicephalus microplus	Wuhan Tick Virus 1 strain X78-2 nucleocapsid (N), ORF2 (ORF2), ORF3 (ORF3), and RNA-dependent RNA polymerase (L) genes, complete cds
**NC_031305.1**	*Rhabdoviridae*	Haemaphysalis hystricis	Yongjia Tick Virus 2 strain YJ1-2 nucleocapsid (N), putative phosphoprotein (ORF2), matrix protein (M), glycoprotein (G), and RNA-dependent RNA polymerase (L) genes, complete cds
**NC_031225.1**	*Rhabdoviridae*	Hyalopterus pruni	Wuhan Insect virus 4 strain YCYC03 nucleocapsid (N), phosphoprotein (P), 4b protein (4b), putative matrix protein (ORF4), glycoprotein (G), and RNA-dependent RNA polymerase (L) genes, complete cds
**NC_031227.1**	*Rhabdoviridae*	Hyalopterus pruni	Wuhan Insect virus 5 strain YCYC02 nucleocapsid (N), phosphoprotein (P), 4b protein (4b), matrix protein (M), glycoprotein (G), and RNA-dependent RNA polymerase (L) genes, complete cds
**NC_031232.1**	*Rhabdoviridae*	Hyalopterus pruni	Wuhan Insect virus 6 strain SXCC01-1 nucleocapsid (N), phosphoprotein (P), 4b protein (4b), matrix protein (M), glycoprotein (G), and RNA-dependent RNA polymerase (L) genes, complete cds
**NC_031240.1**	*Rhabdoviridae*	Hippoboscidae	Wuhan Louse Fly Virus 10 strain BFJSC-8 nucleocapsid (N), putative phosphoprotein (ORF2), matrix protein (M), glycoprotein (G), and RNA-dependent RNA polymerase (L) genes, complete cds
**NC_031278.1**	*Rhabdoviridae*	Musca domestica	Wuhan Fly Virus 2 strain SYY1-3 nucleocapsid (N), ORF2 (ORF2), X protein (X), matrix protein (M), glycoprotein (G), and RNA-dependent RNA polymerase (L) genes, complete cds
**NC_031282.1**	*Rhabdoviridae*	Musca domestica	Wuhan House Fly Virus 1 strain SYY2-4 nucleocapsid (N), putative phosphoprotein (ORF2), putative X protein (ORF3), matrix protein (M), glycoprotein (G), and RNA-dependent RNA polymerase (L) genes, complete cds
**NC_031283.1**	*Rhabdoviridae*	Musca domestica	Wuhan House Fly Virus 2 strain SYY4-5 ORF1 (ORF1), ORF2 (ORF2), ORF3 (ORF3), glycoprotein (G), and RNA-dependent RNA polymerase (L) genes, complete cds
**NC_031215.1**	*Rhabdoviridae*	Musca domestica	Shayang Fly Virus 2 strain SYY1-8 nucleocapsid (N), ORF2 (ORF2), X protein (X), matrix protein (M), glycoprotein (G), and RNA-dependent RNA polymerase (L) genes, complete cds
**NC_031216.1**	*Rhabdoviridae*	Chrysomya megacephala	Shayang Fly Virus 3 strain SYY1-1 ORF1 (ORF1), ORF2 (ORF2), ORF3 (ORF3), glycoprotein (G), and RNA-dependent RNA polymerase (L) genes, complete cds

**NC_031079.1**	*Rhabdoviridae*	Hyalomma asiaticum	Bole Tick Virus 2 strain BL076 nucleocapsid (N), putative phosphoprotein (ORF2), matrix protein (M), glycoprotein (G), and RNA-dependent RNA polymerase (L) genes, complete cds
**NC_028484.1**	*Rhabdoviridae*	Culex bitaeniorhynchus	Tongilchon virus 1 strain A12.2676/ROK/2012, complete genome
**NC_028255.1**	*Rhabdoviridae*		Cocal virus Indiana 2, complete genome
**NC_028246.1**	*Rhabdoviridae*	Bos taurus	Adelaide River virus isolate DPP61, complete genome
**NC_028231.1**	*Rhabdoviridae*	Thunbergia alata	Datura yellow vein virus, complete genome
**NC_028232.1**	*Rhabdoviridae*	Culicoides austropalpalis	Walkabout Creek virus isolate CS1056, complete genome
**NC_028234.1**	*Rhabdoviridae*	Psychodidae	Santa barbara virus strain AR775619, complete genome
**NC_028236.1**	*Rhabdoviridae*	Eidolon helvum	Kumasi rhabdbovirus, complete genome
**NC_028237.2**	*Rhabdoviridae*	Medicago sativa	Alfalfa dwarf virus isolate Manfredi, complete genome
**NC_028239.1**	*Rhabdoviridae*	Bos taurus	Koolpinyah virus isolate DPP819, complete genome
**NC_028241.1**	*Rhabdoviridae*	Mansonia uniformis	Yata virus isolate DakArB 2181, complete genome
**NC_028244.1**	*Rhabdoviridae*		Barley yellow striate mosaic virus strain Hebei, complete genome
**NC_026798.1**	*Rhabdoviridae*		Black grass varicosavirus-like virus segment RNA 2
**NC_026801.1**	*Rhabdoviridae*		Black grass varicosavirus-like virus segment RNA 1
**NC_025385.1**	*Rhabdoviridae*		Khujand lyssavirus, complete genome
**NC_025387.1**	*Rhabdoviridae*	Scophthalmus maximus	Scophthalmus maximus rhabdovirus, complete genome
**NC_025389.1**	*Rhabdoviridae*	Agapanthus	Eggplant mottled dwarf virus isolate Agapanthus, complete genome
**NC_025391.1**	*Rhabdoviridae*	Cryptoblepharus virgatus	Almpiwar virus isolate MRM4059, complete genome
**NC_025393.1**	*Rhabdoviridae*	Culicidae	Arboretum virus isolate Lo-121, complete genome
**NC_025394.1**	*Rhabdoviridae*	Culicidae	Perinet virus, complete genome
**NC_025395.1**	*Rhabdoviridae*	Ochlerotatus fulvus	Puerto Almendras virus isolate LO-39, complete genome
**NC_025396.1**	*Rhabdoviridae*	Bos taurus	Kimberley virus isolate CS368, complete genome
**NC_025397.1**	*Rhabdoviridae*	Bos taurus	Coastal Plains virus strain DPP53, complete genome
**NC_025399.1**	*Rhabdoviridae*	Culex edwardsi	Oak-Vale virus strain CSIRO 1342, complete genome
**NC_025400.1**	*Rhabdoviridae*	Mansonia uniformis	Malakal virus isolate SudAr 1169-64, complete genome
**NC_025401.1**	*Rhabdoviridae*	Gallus gallus	Sunguru virus isolate Ug#41, complete genome
**NC_025405.1**	*Rhabdoviridae*		Niakha virus isolate DakArD 88909, complete genome
**NC_025406.1**	*Rhabdoviridae*	Lagenorhynchus albirostris	Dolphin rhabdovirus isolate pxV1 1992, complete genome
**NC_025382.1**	*Rhabdoviridae*	Spodoptera frugiperda	Spodoptera frugiperda rhabdovirus isolate Sf, complete genome
**NC_025384.1**	*Rhabdoviridae*	Culex tritaeniorhynchus	Culex tritaeniorhynchus rhabdovirus RNA, complete genome, strain: TY
**NC_025340.1**	*Rhabdoviridae*	Amblyomma americanum	Long Island tick rhabdovirus strain LS1, complete genome
**NC_025341.1**	*Rhabdoviridae*	Macronycteris commersoni	Fikirini bat rhabdovirus isolate KEN352, complete genome
**NC_025342.1**	*Rhabdoviridae*	Amblyomma	Kolente virus isolate DakAr K7292, complete genome
**NC_025353.1**	*Rhabdoviridae*	Equus asinus x Equus caballus	Vesicular stomatitis Alagoas virus Indiana 3, complete genome
**NC_025356.1**	*Rhabdoviridae*	Esox lucius	Pike fry rhabdovirus isolate F4, complete genome
**NC_025358.1**	*Rhabdoviridae*	Bos taurus	Berrimah virus strain DPP 63, complete genome
**NC_025359.1**	*Rhabdoviridae*	Culex decens	Moussa virus isolate C23, complete genome

**NC_025362.1**	*Rhabdoviridae*	Sabethes intermedius	Xiburema virus isolate XIBV/BE AR 362159, complete genome
**NC_025364.1**	*Rhabdoviridae*	Ochlerotatus campestris	Malpais Spring virus strain 85-488NM, complete genome
**NC_025365.1**	*Rhabdoviridae*	Macronycteris commersoni	Shimoni bat virus, complete genome
**NC_025371.1**	*Rhabdoviridae*	Tinca tinca	Tench rhabdovirus S64, complete genome
**NC_025376.1**	*Rhabdoviridae*	Ctenopharyngodon idella	Grass carp rhabdovirus V76, complete genome
**NC_025377.1**	*Rhabdoviridae*		West Caucasian bat virus, complete genome
**NC_025378.1**	*Rhabdoviridae*		Yug Bogdanovac virus, complete genome
**NC_025251.1**	*Rhabdoviridae*	Myotis nattereri	Bokeloh bat lyssavirus isolate 21961, complete genome
**NC_025255.1**	*Rhabdoviridae*		Maraba virus from Brazil, complete genome
**NC_024473.1**	*Rhabdoviridae*	Bos taurus	Vesicular stomatitis New Jersey virus isolate NJ1184HDB, complete genome
**NC_022755.1**	*Rhabdoviridae*	Eptesicus fuscus	American bat vesiculovirus TFFN-2013 isolate liver2008, complete genome
**NC_022580.1**	*Rhabdoviridae*	Drosophila obscura	Drosophila obscura sigma virus 10A, complete genome
**NC_022581.1**	*Rhabdoviridae*	Anguilla anguilla	Eel Virus European X complete genome, viral cRNA, isolate 153311
**NC_020803.1**	*Rhabdoviridae*	Perca fluviatilis	Perch perhabdovirus isolate PRV nucleoprotein (N) gene, nucleocapsid (N) gene, complete cds
**NC_020804.1**	*Rhabdoviridae*	Culicoides brevitarsis	Tibrogargan virus strain CS132, complete genome
**NC_020805.1**	*Rhabdoviridae*	Homo sapiens	Chandipura virus isolate CIN 0451, complete genome
**NC_020806.1**	*Rhabdoviridae*	Phlebotomus papatasi	Isfahan virus N gene, P gene, M gene, G gene and L gene, genomic RNA
**NC_020807.1**	*Rhabdoviridae*	Eidolon helvum	Lagos bat virus isolate 0406SEN, complete genome
**NC_020808.1**	*Rhabdoviridae*		Aravan virus, complete genome
**NC_020809.1**	*Rhabdoviridae*		Irkut virus, complete genome
**NC_020810.1**	*Rhabdoviridae*	Homo sapiens	Duvenhage virus isolate 86132SA, complete genome
**NC_018629.1**	*Rhabdoviridae*	Civettictis civetta	Ikoma lyssavirus, complete genome
**NC_018381.2**	*Rhabdoviridae*	Diospyros kaki	Persimmon virus A viral cRNA, complete genome, clone: Kaki13-14
**NC_017685.1**	*Rhabdoviridae*	Mansonia uniformis	Obodhiang virus, complete genome
**NC_017714.1**	*Rhabdoviridae*	Culicoides	Kotonkan virus, complete genome
**NC_016136.1**	*Rhabdoviridae*		Potato yellow dwarf virus, complete genome
**NC_013955.1**	*Rhabdoviridae*		Ngaingan virus, complete genome
**NC_011639.1**	*Rhabdoviridae*	Culicoides austropalpalis	Wongabel virus, complete genome
**NC_011568.1**	*Rhabdoviridae*		Lettuce big-vein associated virus segment 2, complete genome
**NC_011532.1**	*Rhabdoviridae*	Lactuca sativa	Lettuce yellow mottle virus, complete genome
**NC_009609.1**	*Rhabdoviridae*		Orchid fleck virus genomic RNA, segment RNA 2, complete sequence
**NC_009527.1**	*Rhabdoviridae*	Eptesicus serotinus	European bat lyssavirus 1, complete genome
**NC_009528.2**	*Rhabdoviridae*	Homo sapiens	European bat lyssavirus 2 isolate RV1333, complete genome
**NC_008514.1**	*Rhabdoviridae*	Siniperca chuatsi	Siniperca chuatsi rhabdovirus, complete genome
**NC_007642.1**	*Rhabdoviridae*	Allium sativum	Lettuce necrotic yellows virus, complete genome
**NC_007020.1**	*Rhabdoviridae*		Tupaia virus, complete genome
**NC_006942.1**	*Rhabdoviridae*	Colocasia esculenta	Taro vein chlorosis virus, complete genome
**NC_006429.1**	*Rhabdoviridae*		Mokola virus, complete genome
**NC_005974.1**	*Rhabdoviridae*		Maize fine streak virus, complete genome

**NC_005975.1**	*Rhabdoviridae*		Maize mosaic virus, complete genome
**NC_005093.1**	*Rhabdoviridae*	Oncorhynchus mykiss	Hirame rhabdovirus, complete genome
**NC_002526.1**	*Rhabdoviridae*		Bovine ephemeral fever virus, complete genome
**NC_002251.1**	*Rhabdoviridae*		Northern cereal mosaic virus, complete genome
**NC_000903.1**	*Rhabdoviridae*	Channa striata	Snakehead rhabdovirus complete genome
**NC_003243.1**	*Rhabdoviridae*		Australian bat lyssavirus, complete genome
**NC_000855.1**	*Rhabdoviridae*		Viral hemorrhagic septicemia virus Fil3, complete genome
**NC_003746.1**	*Rhabdoviridae*		Rice yellow stunt virus, complete genome
**NC_002803.1**	*Rhabdoviridae*	Cyprinus carpio	Spring viraemia of carp virus, complete genome
**NC_001652.1**	*Rhabdoviridae*	Oncorhynchus tshawytscha	Infectious hematopoietic necrosis virus, complete genome
**NC_001615.3**	*Rhabdoviridae*	Nicotiana x edwardsonii	Sonchus yellow net virus complete genome
**NC_001542.1**	*Rhabdoviridae*		Rabies virus, complete genome
**OZ077875.1**	*Rhabdoviridae*	Onchocerca volvulus	Onchocerca volvulus RNA Virus 1 isolate missing: third party data genome assembly, chromosome: OvRV1
**KP688058.1**	*Flaviviridae*	Culex	Mercadeo virus isolate ER-M10, complete genome
**KT192549.1**	*Flaviviridae*		Parramatta River virus isolate 92-B115745, complete genome
**AB981186.1**	*Flaviviridae*	Culex	Mosquito flavivirus gene for polyprotein, complete cds, strain: YDFV/Oct/2013
**KF917536.1**	*Flaviviridae*	Formicarius analis	Cacipacore virus strain BeAn 3276000, complete genome
**KJ469370.1**	*Flaviviridae*	Cynopterus brachyotis	Batu Cave virus strain P70-1459, complete genome
**KJ469371.1**	*Flaviviridae*	Sigmodon hispidus	Jutiapa virus strain JG-128, complete genome
**KJ469372.1**	*Flaviviridae*	Cynopterus brachyotis	Phnom Penh bat virus strain 30834_A38, complete genome
**KF815939.1**	*Flaviviridae*	Ixodes uriae	Tyuleniy virus strain LEIV-6C polyprotein gene, complete cds
**KC464457.1**	*Flaviviridae*	Culex tritaeniorhynchus	Mosquito flavivirus isolate LSFlaviV-A20-09, complete genome
**KC505248.1**	*Flaviviridae*	Coquillettidia xanthogaster	Palm Creek virus isolate 56 polyprotein gene, complete cds
**JQ268258.1**	*Flaviviridae*	Culicidae	Hanko virus polyprotein gene, complete cds
**JX236040.3**	*Flaviviridae*		Ntaya virus isolate IPDIA, complete genome
**HE574574.1**	*Flaviviridae*	Culex theileri	Culex theileri flavivirus RP-2011 gene for viral polyprotein, genomic RNA, isolate 178
**JF895923.2**	*Flaviviridae*	Anatidae	Tembusu virus strain JS804, complete genome
**DQ859056.1**	*Flaviviridae*		Banzi virus strain SAH 336 polyprotein gene, complete cds
**DQ859057.1**	*Flaviviridae*		Bouboui virus strain DAK AR B490 polyprotein gene, complete cds
**DQ859060.1**	*Flaviviridae*		Edge Hill virus strain YMP 48 polyprotein gene, complete cds
**DQ859062.1**	*Flaviviridae*		Saboya virus strain Dak AR D4600 polyprotein gene, complete cds
**DQ859065.1**	*Flaviviridae*		Uganda S virus polyprotein gene, complete cds
**DQ859066.1**	*Flaviviridae*		Jugra virus strain P-9-314 polyprotein gene, complete cds
**DQ859067.1**	*Flaviviridae*		Potiskum virus strain IBAN 10069 polyprotein gene, complete cds
**GQ165809.2**	*Flaviviridae*	Mansonia africana	Nakiwogo virus strain Uganda08 polyprotein gene, partial cds
**AB488408.1**	*Flaviviridae*	Aedes albopictus	Aedes flavivirus genomic RNA, complete genome, strain: Narita-21
**EU707555.1**	*Flaviviridae*		Wesselsbron virus strain SAH177, complete genome
**FJ644291.1**	*Flaviviridae*	Culex tritaeniorhynchus	Quang Binh virus isolate VN180, complete genome
**AB377213.1**	*Flaviviridae*	Culex pipiens	Culex flavivirus genomic RNA, complete genome, strain: NIID-21-2

**DQ837641.1**	*Flaviviridae*	Chiroptera	Entebbe bat virus strain UgIL-30, complete genome
**DQ837642.1**	*Flaviviridae*	Culicidae	Sepik virus strain MK7148, complete genome
**DQ525916.1**	*Flaviviridae*		St. Louis encephalitis virus strain Kern217, complete genome
**DQ235144.1**	*Flaviviridae*		Meaban virus from France polyprotein gene, complete cds
**DQ235145.1**	*Flaviviridae*		Gadgets Gully virus from Australia polyprotein gene, complete cds
**DQ235146.1**	*Flaviviridae*		Kadam virus from Uganda polyprotein gene, complete cds
**DQ235149.1**	*Flaviviridae*		Royal Farm virus from Afghanistan polyprotein gene, complete cds
**DQ235150.1**	*Flaviviridae*		Saumarez Reef virus from Australia polyprotein gene, complete cds
**DQ235151.1**	*Flaviviridae*		Turkish sheep encephalitis virus polyprotein gene, complete cds
**DQ235152.1**	*Flaviviridae*		Spanish sheep encephalitis virus polyprotein gene, complete cds
**DQ235153.1**	*Flaviviridae*		Greek goat encephalitis virus polyprotein gene, complete cds
**AY323490.1**	*Flaviviridae*		Kyasanur forest disease virus polyprotein gene, complete cds
**AY632535.2**	*Flaviviridae*	Simiiformes	Zika virus strain MR 766, complete genome
**AY632536.4**	*Flaviviridae*		Bussuquara virus strain BeAn 4073, complete genome
**AY632539.4**	*Flaviviridae*		Ilheus virus strain Original, complete genome
**AY632540.2**	*Flaviviridae*	Culicidae	Kedougou virus strain DakAar D1470, complete genome
**AY632541.4**	*Flaviviridae*		Kokobera virus strain AusMRM 32, complete genome
**AY632545.2**	*Flaviviridae*	Culicidae	Bagaza virus strain DakAr B209, complete genome
**AY453411.1**	*Flaviviridae*		Usutu virus strain Vienna 2001 from Austria, complete genome
**AY193805.1**	*Flaviviridae*		Omsk hemorrhagic fever virus strain Bogoluvovska, complete genome
**AB114858.1**	*Flaviviridae*	Chiroptera	Yokose virus genomic RNA, complete genome, strain:Oita 36
**AY149905.1**	*Flaviviridae*		Kamiti River virus isolate SR-82 polyprotein precursor, gene, complete cds
**AJ299445.1**	*Flaviviridae*		Montana myotis leukoencephalitis virus complete genomic RNA
**AJ242984.1**	*Flaviviridae*		Modoc virus genomic RNA for polyprotein gene
**AF311056.1**	*Flaviviridae*		Deer tick virus strain ctb30 polyprotein gene, complete cds
**AF331718.1**	*Flaviviridae*	Homo sapiens	Alkhurma virus strain 1176 polyprotein gene, complete cds
**AF326573.1**	*Flaviviridae*		Dengue virus type 4 strain 814669, complete genome
**AF253419.1**	*Flaviviridae*		Langat virus strain TP21 polyprotein gene, complete cds
**AF160193.1**	*Flaviviridae*		Apoi virus polyprotein gene, complete cds
**AF144692.1**	*Flaviviridae*		Rio Bravo virus strain RiMAR polyprotein gene, complete cds
**AF161266.1**	*Flaviviridae*		Murray Valley encephalitis virus strain MVE-1-51, complete genome
**L40361.3**	*Flaviviridae*		Tick-borne encephalitis virus-Siberian subtype polyprotein gene, complete cds
**U87411.1**	*Flaviviridae*		Dengue virus type 2 (strain 16681) polyprotein mRNA, complete cds
**Y07863.1**	*Flaviviridae*		Louping ill virus, complete genome
**U88536.1**	*Flaviviridae*		Dengue virus type 1 clone 45AZ5, complete genome
**U27495.1**	*Flaviviridae*	Ixodes ricinus	Tick-borne encephalitis virus-European subtype strain Neudoerfl polyprotein gene, complete cds
**M12294.2**	*Flaviviridae*		West Nile virus RNA, complete genome
**M91671.1**	*Flaviviridae*		Flavivirus cell fusing agent polyprotein gene, complete cds
**M18370.1**	*Flaviviridae*		Japanese encephalitis virus (strain JaOArS982), complete genome
**M93130.1**	*Flaviviridae*		Dengue type 3 virus complete genome RNA, complete cds
**L06436.1**	*Flaviviridae*		Powassan virus strain LB, complete genome

**D00246.1**	*Flaviviridae*		Kunjin virus gene for polyprotein (C, prM, E, NS1, NS2A, NS2B, NS3, NS4A, NS4B, NS5), complete cds
**X03700.1**	*Flaviviridae*		Yellow fever virus complete genome, 17D vaccine strain
